# Maternal migraine and offspring ADHD: triangulating the evidence

**DOI:** 10.1186/s12916-026-04692-4

**Published:** 2026-02-14

**Authors:** Yaxin Luo, Christina Dardani, Robyn E. Wootton, Apostolos Gkatzionis, Evie Stergiakouli

**Affiliations:** 1 Population Health Sciences, Bristol Medical School, University of Bristol, Oakfield House, Oakfield Grove, Bristol, UK; 2 Medical Research Council Integrative Epidemiology Unit, Bristol Medical School, University of Bristol, Bristol, UK; 3 Research Department, Lovisenberg Diaconal Hospital, Oslo, Norway; 4 PsychGen Centre for Genetic Epidemiology and Mental Health, Norwegian Institute of Public Health, Oslo, Norway; 5 School of Psychological Science, University of Bristol, Bristol, UK

**Keywords:** Migraine, Attention-deficit/hyperactivity disorder, Prenatal risk factors, Genetic liability, Mendelian randomization

## Abstract

**Background:**

Migraine and attention deficit hyperactivity disorder (ADHD) co-occur more frequently than would be expected by chance. However, little is known about whether there are causal links between maternal migraine during pregnancy and offspring ADHD.

**Methods:**

Using data from the Avon Longitudinal Study of Parents and Children, we performed observational analyses to examine associations between first-trimester maternal migraine and offspring ADHD traits at age 7, with partners’ migraine as a negative control. We complemented this with polygenic risk score (PRS) regression to assess the association of maternal genetic liability to migraine and offspring ADHD. Two-sample Mendelian randomization (MR) was employed to estimate potential causal effects.

**Results:**

Maternal migraine during the first trimester of pregnancy was associated with elevated offspring ADHD traits (OR = 1.59 [1.22, 2.06]) while evidence was weak for partner’s migraine (OR = 1.31 [0.95, 1.82]). Maternal PRS for migraine was associated with increased offspring ADHD traits at age 7 (OR = 1.21 [1.11, 1.32]). MR analyses provided limited evidence of a causal effect of migraine genetic liability on ADHD (OR_IVW_ = 0.96 [0.92, 1.01]). There was weak evidence suggesting a potential causal effect of ADHD genetic liability on migraine (OR_IVW_ = 1.08 [1.02, 1.13]).

**Conclusions:**

Our findings do not support a direct intrauterine causal effect of maternal migraine on offspring ADHD traits. Instead, the observed associations might reflect shared genetic overlap between migraine and ADHD. Future studies should characterize the shared genetic architecture underlying migraine-ADHD links, distinguishing pleiotropic effects from mediated pathways.

**Supplementary Information:**

The online version contains supplementary material available at 10.1186/s12916-026-04692-4.

## Background

Migraine is a chronic neurological condition which can negatively influence quality of life as well as employment and income [1]. The global prevalence of migraine in high-income countries is 14% [2] and migraine is more common in women than men of all ages [3, 4]. During fertile years, women experience a higher incidence and prevalence of migraines compared to women of non-reproductive age, suggesting that female sex hormones may influence the heightened burden of migraine in women [5].

It is well documented that migraine frequently co-occurs with various psychiatric and neurodevelopmental conditions, including depression [6], bipolar spectrum disorder [7], and attention deficit hyperactivity disorder (ADHD) [8]. ADHD is of particular interest because it typically manifests in early childhood, has a prevalence of up to 7.6% in children aged 3 to 12 years [9], and substantially affects children’s developmental and educational outcomes [10]. Genetic study also indicated that migraine and ADHD share common heritability [11].

While cross-sectional studies have provided valuable insights on the links between migraine and ADHD [12], much less is known about the cross-generational association between maternal migraine and offspring ADHD. Registry-based research provides preliminary evidence: a Danish cohort (*n* = 2,069,785) reported that children of mothers with migraine-related hospital admissions or outpatient clinic visits had a 22% higher risk of developing psychiatric disorders [13]. In line with this, a Taiwanese study (*n* = 250,217) suggested that parental migraine history was associated with a 37% higher risk of ADHD in offspring [14]. Yet, these studies may underestimate maternal migraine prevalence—only 2.5% in the Danish dataset [13], and are likely to capture predominantly severe cases, raising concerns about misclassification.

The mechanisms underlying this association remain unclear, but both shared genetic factors and intrauterine environment may play a role. Maternal migraine has been linked to pregnancy-related hypertension [15], which increases the risk of preterm birth, a factor associated with disrupted brain maturation and elevated ADHD risk [16]. This pathway is of particular clinical interest because it represents a potentially modifiable risk factor. Alternatively, genetic confounding may explain the association: migraine and ADHD share common genetic determinants (*r*_*g*_ = 0.26, *P* = 8.81 × 10^−8^) [17], and pleiotropic genetic effects may predispose individuals to both conditions. In addition, ADHD could be contributing to the increased risk of migraine [18], raising the possibility that maternal ADHD contributes to the transmission of risk across generations. Unlike intrauterine mechanisms, these genetic pathways would not be directly modifiable, underscoring the importance of disentangling intrauterine effects from genetic transmission.

In this study, we triangulate evidence on maternal migraine and offspring ADHD by integrating observational analyses and negative control analyses in parent–child trios, intergenerational polygenic risk score (PRS) analyses, and bidirectional Mendelian randomization (MR), to evaluate potential causal mechanisms.

## Methods

### Observational associations

#### The Avon Longitudinal Study of Parents and Children (ALSPAC)

Pregnant women resident in Avon, UK, with expected dates of delivery between 1 st April 1991 and 31 st December 1992 were invited to take part in the study. A total of 20,248 pregnancies have been identified as being eligible and the initial number of pregnancies enrolled was 14,541. Of the initial pregnancies, there was a total of 14,676 foetuses, resulting in 14,062 live births and 13,988 children who were alive at 1 year of age. Due to efforts made when the children were around 7 years old to recruit additional eligible pregnancies who had not originally joined the study, the sample size increased. The total sample size for analyses using any data collected after the age of 7 is therefore 15,447 pregnancies, resulting in 15,658 foetuses. Of these, 14,901 children were alive at 1 year of age. The details of the cohort have been presented in previous work [19, 20]. A total of 12,113 G0 partners have been in contact with the study by providing data and/or formally enrolling when this started in 2010. A total of 3807 G0 partners are currently enrolled. Data on the children and their parents were collected through questionnaire completion, attending clinics in person, and genotyping. The study website contains details of all the data available through a fully searchable data dictionary: http://www.bristol.ac.uk/alspac/researchers/our-data. Informed consent for the use of data collected via questionnaires and clinics was obtained from participants following the recommendations of the ALSPAC Ethics and Law Committee at the time http://www.bristol.ac.uk/alspac/researchers/research-ethics/.

For families with more than one child, only the eldest child was included.

#### Exposure: parental migraine during pregnancy

At 12 weeks of gestation, mothers were asked through a questionnaire: ‘Have you ever had any of the following problems?’ with multiple options. For migraine, mothers could choose ‘No never’, ‘Yes in past, not now’, ‘Yes had it recently’, and ‘Don’t know’. Participants who answered ‘No never’ were categorized as ‘No’. Participants who answered ‘Yes in past, not now’ were categorized as having ‘History of migraine’. Participants who answered ‘Yes had it recently’ were classified as ‘migraine during the first trimester of pregnancy’. Partners were asked the same question at the same time point and we followed the same migraine variable definition with the pregnant mothers. Mothers who did not reply to the questionnaire or answered ‘Don’t know’ were excluded from the main analysis.

#### Outcome: child ADHD traits at age 7

In line with previous work in the ALSPAC cohort [21], we used the hyperactivity subscale (score range 0–10 based on 5 items) of the mother-reported Strength and Difficulties Questionnaire (SDQ) [22], administered when the children were 7 years old. Children were considered as presenting ADHD traits if they scored 7 or higher on the hyperactivity subscale.

#### Covariates

In analyses investigating the observational associations between maternal migraine and ADHD in their offspring, several confounders were considered: maternal baseline characteristics (highest educational attainment, social class collected at 32 weeks of gestation, marital status), maternal lifestyle (maternal alcohol consumption during the first trimester of pregnancy), maternal mental health condition assessed at 18 weeks of gestation (Edinburgh Postnatal Depression Score [EPDS] and maternal psychiatric symptomatology measured by total Crown-Crisp Experiential Index [CCEI]). More details about these variables are presented in Additional file 1: Note 1 [23–36].

#### Negative control analyses

We compared the associations between maternal prenatal migraine and offspring ADHD traits to the association of partner’s migraine during pregnancy with offspring ADHD traits. If an intrauterine effect of migraine on offspring ADHD is present, we would expect stronger associations with maternal prenatal migraine and much weaker associations with partner’s migraine because only maternal prenatal exposures can have direct biological effects on the foetus [37]. Since we do not account for a biological relationship between father and child, some partner-child pairs may not share a genetic background, leading to an underestimation of the genetic contribution to the association and potentially biasing the negative control comparison towards the null.

#### Statistical analyses

Three models were fitted to estimate the association between maternal migraine and offspring ADHD trait. Model 1 was a univariate model; model 2 was adjusted for maternal highest educational attainment, social class (32 weeks’ gestation), marital status, and alcohol consumption during the first trimester of pregnancy; the fully adjusted model 3 was further adjusted for maternal EPDS and maternal psychiatric symptomatology measured by total CCEI at 18 weeks’ gestation.

In negative control analysis, we restricted sample to families consisting of mother-partner-child trios (same sample size in two models) and applied the same models as in our main analyses. We used the same maternal covariates in both maternal and paternal models to capture shared familial and socioeconomic confounding, as parental exposure are highly correlated through assortative mating and shared environment [38].

### Polygenic risk scores

#### Genotyping, SNP selection, and PRS calculation

To generate a common variant PRS for migraine in ALSPAC mothers, we utilized the latest fully publicly available GWAS for migraine at the time of analysis (48,975 cases and 540,381 controls). This genome-wide meta-analysis included four studies: IHGC 2016, UKBB, GeneRISK, and HUNT. Details on the GWAS can be found in the original publication [39, 40]. Individual-level genotype data from the ALSPAC cohort were used as the target sample. A total of 10,015 women (G0 mothers from the ALSPAC cohort) were genotyped using the Illumina human660-quad array which contains 557,124 SNP markers. After quality control, 9048 subjects and 526,688 SNPs remained. The genotype data of mothers and children were jointly imputed based on the 1000 genomes reference panel (phase 1, version 3), resulting in 8196 eligible G0 mothers with available genotype data after exclusion of related subjects within mothers (details in Additional file 1: Note 2). After removing participants who had withdrawn consent, genotype data were available for 7927 participants.

PRSs for migraine in G0 mothers were estimated in the eligible sample using PLINK 1.9. Genetic variants from the target sample were excluded if: (a) there were allelic mismatches between target and discovery samples; (b) the variants were situated within the major histocompatibility complex (MHC) region due to their extreme LD complexity, pleiotropic effects, and unreliable imputation, which can introduce bias and confounding (chromosome 6, from base pair position 25,000,000 to 34,000,000, according to the hg19 build 37 reference genomes) [41]. After filtering for variants with imputation quality > 0.8 and minor allele frequency > 0.01, clumping was performed with an *r*^2^ parameter of 0.25 and a physical distance threshold of 500 kB. Additionally, palindromic SNPs were excluded before calculating the PRS. Eight *P* value thresholds for SNP inclusion were then implemented to estimate PRS for migraine: 0.5, 0.1, 0.05, 0.005, 0.001, 1 × 10^−5^, 5 × 10^−6^, and 5 × 10^−8^. Cox and Snell *R*^2^ was calculated for each PRS [42], and the PRS threshold that explained the greatest variance for migraine during the first trimester, compared to individuals who had never experienced migraines, was selected for the main analysis. As a supplementary analysis, we tested whether ADHD-PRS, derived from the latest ADHD GWAS at multiple* P* value thresholds (5 × 10^−8^ to 0.5), were associated with phenotypic migraine in ALSPAC mothers.

#### Statistical analyses

We conducted logistic regression to examine the associations between maternal PRS-migraine and offspring ADHD traits at age 7, additionally adjusting for the first ten maternal genetic principal components (PCs). We used multiple PRS generated with different *P* value thresholds in sensitivity analysis to test if results were consistent with the main analysis.

### Two-sample Mendelian randomization

MR is a method that leverages genetic variants as instrumental variables (IVs) to test for causality [43]. MR facilitates the unbiased estimation of causal relationships, given the fulfilment of three key assumptions [44]: (1) relevance, the genetic variants must be robustly associated with the exposure of interest; (2) independence, there should be no confounders of the association of genetic variants with the outcome; (3) exclusion restriction, the genetic variants must influence the outcome only via exposure, without any direct pathway or pleiotropic effects. To validate the first assumption when estimating the causal effect of migraine on ADHD, we selected independent IVs for migraine from the same GWAS as the one we utilized in PRS regression (*P* < 5 × 10^−8^, *r*^2^ < 0.01, 1000 kB) [45]. Although a more recent GWAS with a larger sample size is available, full summary statistics from this dataset were not accessible due to data sharing restrictions related to 23andMe. The GWAS including 23andMe was used in sensitivity analyses to assess robustness. The outcome GWAS was derived from the Psychiatric Genomics Consortium (PGC) for ADHD (38,691 ADHD cases and 186,843 controls) [46].

To assess bidirectional effects, we performed reverse-direction MR using ADHD genetic variants (same PGC GWAS) [46] as exposures and migraine GWAS summary statistics as the outcome [40], applying identical IV selection criteria.

Although our research question is whether maternal migraine might be causing offspring ADHD, we note the absence of maternal-specific migraine GWAS precludes direct genetic instrument selection, which means the MR of migraine on ADHD cannot specifically capture maternal effects on offspring ADHD [47]. Loci identified in GWAS samples of non-pregnant adults may partially reflect the genetic predisposition to migraine during pregnancy, though pregnancy-specific hormonal and vascular modifications could modulate their effects [5]. In sensitivity analysis described below, we explicitly tested the performance of these migraine IVs for predicting migraine occurring during pregnancy.

#### Statistical analyses

The conventional inverse variance weighted (IVW) method was used as the primary approach [48]. Cochran’s *Q* test was applied to test for heterogeneity [49]. If significant heterogeneity was detected, indicating differences in effect sizes across variants, a random-effects model was applied to account for variability [50]. In addition, we applied various methods to assess the robustness of the results when MR assumptions were violated, including MR-Egger regression, weighted median, weighted mode, and simple mode methods (Additional file 1: Note 3 [51–53]).

#### Sensitivity analyses

We performed sensitivity analyses to firstly test the validity of MR assumptions while ensuring IVs applicability to during-pregnancy migraine. For this, we tested the concordance of effect direction using data from ALSPAC mothers (Ncase = 1041 with first-trimester migraine, Ncontrol = 7732 migraine-free controls). We compared the effect direction of these pre-selected SNPs between the external GWAS [40] and ALSPAC, retaining only SNPs demonstrating consistent effects.

Because of data access limitations, the IVs used in the main analyses may capture less variance in migraine than those from the full GWAS including 23andMe. We therefore performed sensitivity analyses to test the robustness of the results across different instrument selection strategies. For this, we used two additional sources to identify SNPs associated with maternal migraine during pregnancy: (1) GWAS in primary analysis with 23andMe to enhance statistical power (restricted to genome-wide significant SNPs due to data access restrictions, 102,084 migraine cases and 771,257 controls, *P* < 5 × 10^−8^) [40]; and (2) female-specific GWAS for migraine (22,500 migraine cases and 279,762 controls) to ensure that the genetic instruments were more representative of the study population, as genetic effects on migraine may differ by sex [54]. These analyses were performed using the ‘TwoSampleMR’ package in R 4.1.2 [55].

## Results

### Observational associations between maternal migraine during pregnancy and ADHD traits in the offspring

As shown in Table 1, compared with mothers who had migraine before pregnancy and mothers who experienced migraine during the first trimester, mothers who had never experienced migraine before the first trimester tended to have higher education level, better social class, and lower scores for both depression and psychiatric symptoms.

**Table 1:** Baseline characteristics among mothers with no migraines, history of migraine, and migraine during the first trimester

Variable	All participants (*N* = 12,299)	Never had migraine (*N* = 6856, 55.7%)	History of migraine (*N* = 3539, 28.8%)	Migraine during the first trimester (*N* = 1904, 15.5%)	*P* value
Maternal highest educational attainment					4.08 × 10^−15^
A level	2662 (23)	1597 (24.7)	711 (21.4)	354 (19.9)	
CSE/vocational	3319 (28.7)	1759 (27.2)	1001 (30.1)	559 (31.4)	
Degree	1537 (13.3)	964 (14.9)	399 (12)	174 (9.8)	
O level	4055 (35)	2152 (33.3)	1211 (36.5)	692 (38.9)	
Missing	726	384	217	125	
Maternal age when pregnant					8.19 × 10^−9^
Mean (SD)	27.9 (4.8)	28.2 (4.8)	27.6 (4.9)	27.6 (4.8)	
Missing	350	193	94	63	
Maternal social class					0.007
I	566 (5.9)	354 (6.5)	147 (5.4)	65 (4.6)	
II	3038 (31.8)	1774 (32.8)	832 (30.5)	432 (30.6)	
III (non-manual)	4112 (43.1)	2306 (42.6)	1181 (43.3)	625 (44.3)	
III (manual)	728 (7.6)	392 (7.2)	224 (8.2)	112 (7.9)	
IV	909 (9.5)	494 (9.1)	274 (10.1)	141 (10)	
V	197 (2.1)	94 (1.7)	68 (2.5)	35 (2.5)	
Missing	2749	1442	813	494	
Maternal marital status					1.02 × 10^−5^
Never	2126 (17.7)	1122 (16.8)	645 (18.7)	359 (19.4)	
1 st marriage	8388 (70)	4789 (71.6)	2361 (68.5)	1238 (66.9)	
Marriage 2 or 3	780 (6.5)	436 (6.5)	203 (5.9)	141 (7.6)	
Widowed/divorced/separated	696 (5.8)	344 (5.1)	240 (7)	112 (6.1)	
Missing	309	165	90	54	
Maternal alcohol consumption during first trimester					0.86
< 1 glass per week	4672 (39.2)	2614 (39.3)	1352 (39.2)	706 (38.7)	
1 + glass per week	1860 (15.6)	1046 (15.7)	540 (15.7)	274 (15)	
Never	5385 (45.2)	2986 (44.9)	1553 (45.1)	846 (46.3)	
Missing	382	210	94	78	
Maternal EPDS					< 2.2 × 10^−12^
Mean (SD)	7.3 (4.6)	6.8 (4.4)	7.7 (4.7)	8.3 (4.9)	
Missing	1926	1165	512	249	
Maternal CCEI					< 2.2 × 10^−12^
Mean (SD)	13.6 (7.7)	12.4 (7.2)	14.4 (7.7)	16.1 (8.2)	
Missing	1763	1005	480	278	
Offspring hyperactive symptoms at age 7					2.07 × 10^−6^
No	7170 (89.3)	4134 (90.7)	2021 (88.3)	1015 (85.8)	
Yes	861 (10.7)	426 (9.3)	267 (11.7)	168 (14.2)	
Missing	4268	2296	1251	721	

Descriptive statistics are presented as counts (%) if not otherwise specified.* P *values compare the three migraine groups (never/history/during first trimester) using *t*-tests (continuous) or chi-square tests (categorical)

*EPDS* Edinburgh Postnatal Depression Score, *CCEI* Crown-Crisp Experiential Index

The baseline characteristics of mothers who completed questionnaires related to migraine status at 12 weeks of gestation, compared to those who did not, are summarized in Additional file 2: Table S1. Mothers who completed the questionnaires (78.6%) were more likely to have higher educational attainment and report a higher occupational social class compared to mothers who did not complete the questionnaire. Additionally, they exhibited lower depression scores, as measured by the EPDS, and lower levels of psychiatric symptomatology, as measured by the CCEI at 18 weeks of gestation.

As presented in Table 2, compared with children whose mothers never experienced migraine, children whose mothers experienced migraine during the first trimester of pregnancy were more likely to present with increased ADHD traits at age 7 (maternal migraine during the first trimester vs. never had migraine: OR = 1.41, 95% CI = 1.12, 1.78; *P* = 0.004; fully adjusted model). Evidence was weaker for maternal migraine history as the estimates attenuated in the fully adjusted model (model 2: OR = 1.26, 95% CI = 1.05, 1.51, *P* = 0.014; fully adjusted model: OR = 1.16, 95% CI = 0.95, 1.41, *P* = 0.147).

**Table 2:** The association between maternal migraine status and offspring ADHD symptoms at age 7

Variable	Cases/total	OR (95% CI)	*P* value
Model 1			
Never had migraine	426/4560	Reference	
History of migraine	267/2288	1.28 (1.09, 1.51)	0.003
Migraine during the first trimester	168/1183	1.61 (1.33, 1.94)	1.20 × 10^−6^
Model 2			
Never had migraine	344/3801	Reference	
History of migraine	213/1858	1.26 (1.05, 1.51)	0.014
Migraine during the first trimester	134/947	1.59 (1.28, 1.97)	2.39 × 10^−5^
Model 3 (fully adjusted model)			
Never had migraine	293/3154	Reference	
History of migraine	184/1588	1.16 (0.95, 1.41)	0.147
Migraine during the first trimester	124/842	1.41 (1.12, 1.78)	0.004

Model 1 is a univariate model; model 2 is adjusted for maternal age at delivery, maternal highest educational attainment, social class, marital status, and alcohol consumption during first trimester; model 3 is further adjusted for maternal depression and neurotic symptoms

### Negative control analyses

**Fig. 1:**
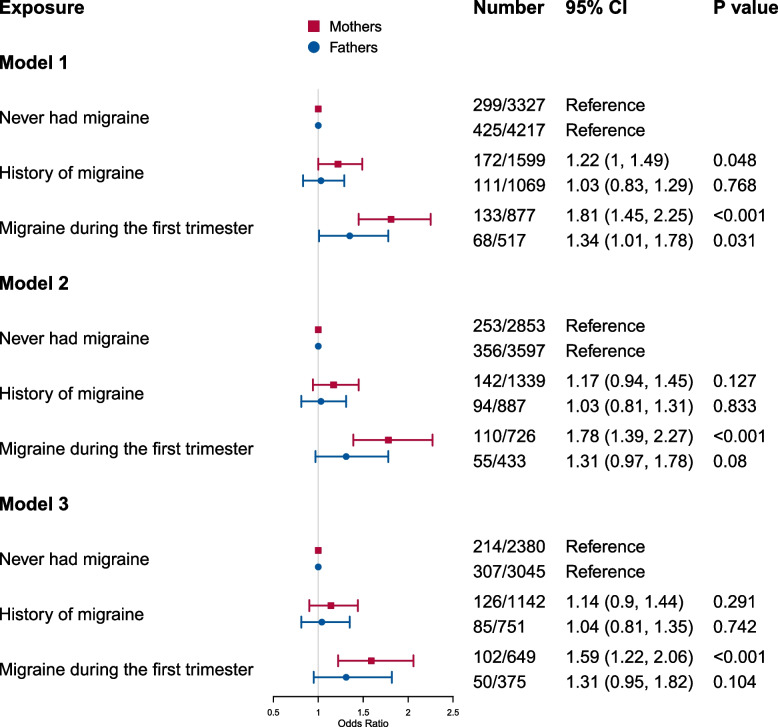
The association between parental migraine status and offspring ADHD traits at age 7 years. Model 1 was a univariate model; model 2 was adjusted for maternal highest educational attainment, social class, marital status, and alcohol consumption during first trimester; model 3 was further adjusted for maternal depression and neurotic symptoms. The associations were conducted in mother-partner-offspring duos

A total of 5803 mother-partner-offspring trios with complete data were included in the negative control analysis. As shown in Fig. 1, after adjusting for the same covariates, there was little evidence for an association between partner’s migraine and offspring ADHD traits at age 7 (history of migraine: OR = 1.04, 95% CI = 0.81, 1.35, *P* = 0.742; migraine during the first trimester: OR = 1.31, 95% CI = 0.95, 1.82, *P* = 0.104; compared with partners who never had migraine). In contrast, maternal migraine during the first trimester remained associated with increased offspring ADHD traits (history of migraine: OR = 1.14, 95% CI = 0.90, 1.44, *P* = 0.291; migraine during the first trimester: OR = 1.59, 95% CI = 1.22, 2.06, *P* = 0.001; compared with mothers who never had migraine).

### Associations between maternal PRS for migraine and ADHD traits in offspring

**Fig. 2:**
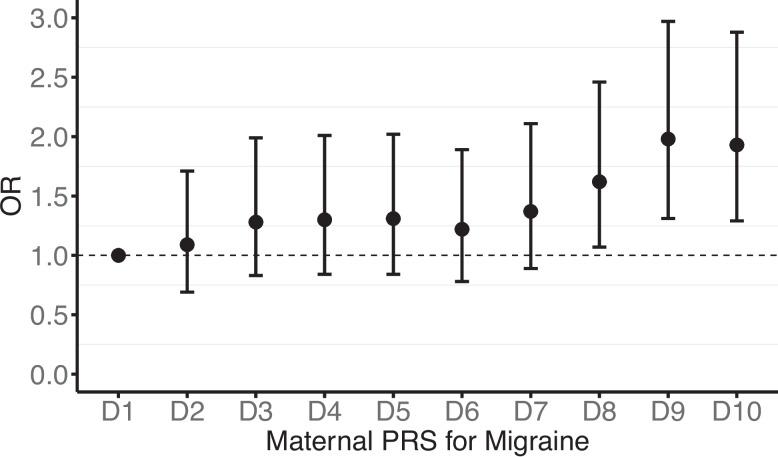
The association between maternal genetic liability to migraine and offspring ADHD traits. Models were adjusted for maternal first ten principal components

The maternal migraine PRS corresponding to the *P* < 0.5 threshold was found to explain more variance of maternal migraine compared to the rest of the thresholds and was therefore used as the primary threshold for subsequent analyses (Additional file 2: Table S2). Using this PRS as a categorical variable (deciles), mothers in the top 30% of genetic liability to migraine had a higher likelihood of their offspring exhibiting ADHD traits at age 7 compared to those in the lowest decile (decile 8: OR = 1.62, 95% CI = 1.07, 2.46, *P* = 0.023; decile 9: OR = 1.98, 95% CI = 1.31, 2.97, *P* = 0.001; decile 10: OR = 1.93, 95% CI = 1.29, 2.88, *P* = 0.001; Fig. 2). When examining PRS constructed at increasingly stringent thresholds (from *P* < 1 × 10^−3^ to genome-wide significant, *P* < 5 × 10^−8^) as continuous predictors, the association with offspring ADHD traits became progressively weaker, with little evidence of association at the most stringent threshold (*P* < 5 × 10^−8^: OR = 1.04, 95% CI = 0.95, 1.14, Additional file 2: Table S2A). We examined the association between migraine and ADHD-PRS across multiple thresholds in mothers from ALSPAC. There was some evidence that higher ADHD-PRS was associated with an increased likelihood of migraine when using more inclusive thresholds (e.g. *P* < 0.05; OR = 1.11, 95% CI = 1.06, 1.17, *P* < 0.001). However, when restricting to genome-wide significant variants (*P* < 5 × 10^−6^), the evidence for association was weaker (OR = 1.04, 95% CI = 0.99, 1.09, *P* = 0.111; Additional file 2: Table S2B).

### Two-sample Mendelian randomization

#### Testing for a causal effect of genetic liability to migraine on ADHD

In our primary analysis, after excluding SNPs that were not available in the outcome GWAS and that were palindromic, 36 SNPs with *F* statistics greater than 10 were used as IVs for migraine in the general population (Additional file 2: Table S3). We found little evidence for a causal effect of genetic liability to migraine on ADHD (OR_IVW_ = 0.96 [0.92, 1.01], *P* = 0.162, Table 3). There was little evidence of bias from horizontal pleiotropy from the MR-Egger intercept (intercept = 0.01, 95% CI = − 0.01, 0.02; Additional file 2: Table S4). As evidence of heterogeneity was identified from Cochran’s *Q* test (Additional file 2: Table S4), a random-effect IVW was implemented (OR_IVW_ = 0.96 [0.92, 1.03], *P* = 0.298, Table 3). All the SNPs passed the Steiger filtering test.

**Table 3:** Bidirectional two-sample Mendelian randomization estimation between migraine and ADHD

Method	OR (95% CI)	*P* value
Forward causation: genetic liability to migraine on ADHD		
Inverse variance weighted^a^	0.96 (0.9, 1.03)	0.298
Inverse variance weighted^b^	0.96 (0.92, 1.01)	0.162
MR-Egger	0.89 (0.73, 1.08)	0.241
Weighted median	0.96 (0.89, 1.04)	0.291
Weighted mode	0.96 (0.86, 1.06)	0.385
Simple mode	0.95 (0.83, 1.08)	0.426
Reverse causation: genetic liability to ADHD on migraine		
Inverse variance weighted^a^	1.08 (1, 1.16)	0.051
Inverse variance weighted^b^	1.08 (1.02, 1.13)	0.003
MR-Egger	0.89 (0.66, 1.21)	0.472
Weighted median	1.08 (1, 1.16)	0.063
Weighted mode	1.19 (1.01, 1.4)	0.052
Simple mode	1.23 (1.01, 1.49)	0.045

^a^Random effect inverse variance weighted model

^b^Fixed effect inverse variance model

#### Robustness check for during-pregnancy effects

In our sensitivity analysis (testing IV validity for during-pregnancy effects), restricting to SNPs (*N* = 13) with consistent directions in the migraine GWAS and the ALSPAC genotype data did not provide evidence of causal effects (OR_IVW_ = 1.03 [0.92, 1.15], *P* = 0.608, Additional file 2: Table S5).

#### Robustness check using sex-stratified and largest GWAS SNPs

In our second sensitivity analysis, a total of 109 and 16 SNPs were extracted from the GWAS for migraine including 23andMe [40] and female-specific GWAS [54], respectively (Additional file 2: Tables S6 and S7). Sensitivity analyses using genome-wide significant SNPs from the larger GWAS including 23andMe, as well as a female-specific GWAS, yielded consistent null findings (Additional file 2: Table S8).

#### Testing for a causal effect of genetic liability to ADHD on migraine

For the reverse direction, we tested if there is a causal effect of genetic liability to ADHD on migraine. Twenty-seven SNP with *F* statistics greater than 10 were extracted as IVs for ADHD (Additional file 2: Table S9). A marginal effect of genetic liability to ADHD increasing migraine risk was suggested by the IVW method (OR = 1.08 [1.02, 1.13], *P* = 0.003) and there were similar results from weighted mode and weighted median (Table 3). We found little evidence for bias from horizontal pleiotropy from MR-Egger intercept (intercept = 0.01, 95% CI = − 0.01, 0.03; Additional file 2: Table S10). As Cochran’s *Q* test identified heterogeneity (Additional file 2: Table S10), we also conducted a random-effect IVW model (OR = 1.08 [1, 1.16], *P* = 0.051, Table 3).

## Discussion

In this study, we utilized a population-based birth cohort to assess the association between maternal migraine during pregnancy and offspring ADHD traits. Maternal migraine during the first trimester of pregnancy was associated with increased offspring ADHD traits at age 7, although estimates were attenuated in negative control analyses. In addition, mothers with higher genetic liability to migraine (as suggested by migraine PRS using a liberal *P* value threshold) were more likely to have children with increased ADHD traits at age 7. Little evidence of a causal effect of genetic liability to migraine on ADHD was identified using MR. In contrast, our analysis revealed evidence supporting an effect of genetic liability to ADHD on migraine.

Our study provides evidence that maternal migraine during the first trimester of pregnancy and maternal genetic liability to migraine might be linked to offspring ADHD traits in early childhood. Negative control analyses indicated overlapping confidence intervals between maternal and partner associations, suggesting that this association is unlikely to be driven primarily by intrauterine effects. Nevertheless, we cannot fully exclude a minor role of intrauterine mechanisms.

Our results extend the literature for the association between parental migraine and offspring ADHD. A case–control familial association study found that migraine was more frequently observed in mothers of children with ADHD than mothers without children with ADHD (69.0% vs. 20.9%) while little evidence was found in fathers (21.0% vs. 14.1%) [56]. Consistently, a prior East Asian cohort study reported a positive association of maternal migraine and offspring ADHD (HR = 1.43 [1.29–1.59]) with non-significant paternal effects (HR = 1.18 [0.99, 1.42]) [14]. Our European population study replicates this pattern, with overlapping parental effect estimates, suggesting limited evidence for parent-of-origin effect in the parental migraine-ADHD association.

The observed association appears to be primarily driven by genetic factors implicated in ADHD and migraine rather than direct intrauterine effects. Two distinct genetic mechanisms could underlie this pattern. First, genetic confounding, in which maternal genetic liability to ADHD influences maternal migraine risk (through maternal ADHD as a phenotypic manifestation) and is simultaneously transmitted to offspring, increasing ADHD risk. Secondly, migraine and ADHD share overlapping genetic variants which could be increasing risk to both ADHD and migraine independently of maternal ADHD. Indeed, previous genome-wide studies have consistently demonstrated genetic overlap between migraine and ADHD, as well as pleiotropic loci implicating common biological pathways [57, 58].

A previous study supported our conclusions as LD score regression indicated that migraine is genetically correlated with ADHD (*r*_*g*_ = 0.26, *P* = 8.81 × 10^−8^) [17]. Our PRS analyses across multiple *P* value thresholds align with this interpretation. Stringent migraine-associated SNPs (*P* < 1 × 10^−3^) showed little effect on offspring ADHD traits, whereas weaker SNPs (*P* < 5 × 10^−3^) showed moderate effects, consistent with weak pleiotropic effects or reverse causation. Two-sample MR using genome-wide significant SNPs (*P* < 5 × 10^−8^) found no evidence of a causal effect of migraine genetic liability on ADHD, further supporting a genetic explanation: if intrauterine effects predominated, these SNPs would be expected to strongly predict offspring ADHD. In addition, our supplementary analyses showed evidence that higher ADHD-PRS is associated with increased risk of migraine in women at more inclusive *P* value threshold, but not at the stringent threshold (*P* < 5 × 10^−6^), consistent with a polygenic overlap rather than driven by a few top loci. These findings are consistent with previous studies using larger samples [59], which also reported associations between ADHD-PRS and migraine, further supporting a shared polygenic basis. Bidirectional MR suggested some causal effect of ADHD genetic liability on maternal migraine. This may reflect that maternal genetic liability to ADHD manifests in behavioural traits, such as smoking [60], which is a risk factor for migraine. Collectively, these findings suggest that shared genetic and familial factors are the main contributors to the observed association between maternal migraine and offspring ADHD, with intrauterine effects likely playing a minor role.

While shared familial factors likely play a primary role, current evidence does not allow us to rule out intrauterine mechanisms entirely. For example, maternal migraine is consistently associated with elevated preterm birth risk (pooled OR = 1.26 [1.21, 1.32]) [61], and preterm birth itself predicts higher ADHD traits in offspring [16]. Neuroimaging evidence indicates that preterm birth alters brain development (e.g. reduced white/grey matter volumes) [62], partially mirroring structural changes individuals observed with ADHD [63]. However, given our MR and PRS regression analyses suggested that genetic transmission is the predominate contributor, these intrauterine pathways likely play a limited role in the observed association.

Strengths of this study include the nature of the prospective design and the triangulation approach that integrates several methodological approaches to examine the relationship between maternal migraine and offspring ADHD traits.

Our findings should be interpreted with caution in light of several limitations. First, due to substantial missing ADHD data in ALSPAC, which is often non-random and related to symptom severity [64], we opted against imputation and analysed available cases only. Thus, results should be interpreted with caution regarding generalizability. Second, maternal migraine was self-reported in the first trimester, which may have led to misclassification if other headaches were inaccurately reported as migraines. Additionally, in the context of the negative control analysis, we could not confirm partner paternity, so potential genetic contributions may have been missed if the partner was not the biological father. Third, our findings should be interpreted in light of potential reporting bias. Although parent report is a key and widely used source for assessing childhood ADHD [65], evidence from paediatric psychology suggests that parental cognitive and emotional characteristics, such as pain catastrophizing, may influence child symptoms reporting [66, 67]. Mothers with migraine may therefore be more likely to notice or report offspring symptoms, potentially inflating observed association.

Fourth, maternal genetic liability to migraine was generated based on the publicly available GWAS which includes ALSPAC (2.3% of total sample). Partial sample overlap may lead to some inflation of PRS estimation, likely explaining the relatively high pseudo-*R*^2^ observed. Importantly, this does not affect the interpretation of the main results: the key finding—that maternal polygenic liability to migraine is associated with offspring ADHD traits—remains valid. These estimates should be interpreted cautiously and not as absolute measures of predictive performance. Fifth, we only included common variants to generate migraine PRS. A recent study highlighted that rare variants show large and informative effects on migraine and different subtypes, such as migraine with aura and migraine without aura can have distinct mechanisms [68]. Sixth, it is worth noting that we performed two-sample MR using publicly available data which were not specific to migraine during pregnancy and due to the small sample size of mother–child pairs in ALSPAC, we did not have power to conduct MR with maternal and offspring genetic data nor within-family MR [69]. Finally, a formal mediation analysis to investigate potential intrauterine pathways was not performed. Given our sample size and the primary aim of distinguishing intrauterine effects from inherited genetic liability, such an analysis would have had limited power and interpretability. Future studies with larger cohorts specifically designed to explore potential mediating pathways could provide additional insights.

## Conclusions

This study provides information regarding the link between maternal migraine (during pregnancy) and offspring ADHD traits (during childhood) by using a birth cohort based in the UK. Our findings suggest that the identified links could be at least partially due to genetic overlap between the two conditions. Investigating the biological pathways that are shared between them might point to potential interventions which could benefit both migraine sufferers and ADHD individuals.

## Supplementary Information

Additional file 1. Notes 1–3. Note 1 Baseline characteristic assessment in Avon Longitudinal Study of Parents and Child. Note 2 Details on genotyping in Avon Longitudinal Study of Parents and Child. Note 3 Mendelian randomizationmethod.

Additional file 2. Tables S1–S10. Table S1 The baseline characteristics for mothers who completed and did not complete questionnaires collected at 12 weeks’ gestation. Table S2 Associations between polygenic risk scoresand migraine/ADHD traits. Table S2A Association between maternal migraine PRS and offspring ADHD traits. Table S2B Association between ADHD-PRS and migrainein mothers from ALSPAC. Table S3 Harmonized SNP data for two-sample MR to estimate the causal effect of genetic liability to migraine on ADHD. Table S4 Heterogeneity and horizontal pleiotropy test for IVs. Table S5 Estimated causal effect of genetic liability to migraine on ADHD using IVs validated in ALSPAC data during pregnancy. Table S6 Harmonized SNP data for two-sample MR to estimate the causal effect of genetic liability to migraine on ADHD. Table S7 Harmonized SNP data for two-sample MR to estimate the causal effect of genetic liability of migraine on ADHD. Table S8 Secondary sensitivity analysis—MR estimation for causal effect of genetic liability to migraine on ADHD. Table S9 Harmonized SNP data for two-sample MR to estimate the causal effect of genetic liability to ADHD on migraine. Table S10 Heterogeneity and horizontal pleiotropy test for IVs.

## Data Availability

Data from ALSPAC is accessible to all researchers upon submitting an application. The GWAS summary data can be download from the corresponding paper. The codes used in this study are available upon request by contacting the corresponding authors.

## Abbreviations

ALSPAC: The Avon Longitudinal Study of Parents and Children
ADHD: Attention deficit hyperactivity disorder
PRS: Polygenic risk score
MR: Mendelian randomization
IV: Instrumental variable
SDQ: Strength and Difficulties Questionnaire
EPDS: Edinburgh Postnatal Depression Score
CCEI: Crown-Crisp Experiential Index
MHC: Major histocompatibility complex
GWAS: Genome-wide association studies
SNP: Single nucleotide polymorphism
PC: Principal components
PGC: Psychiatric Genomics Consortium
IVW: Inverse variance weighted
