# Supplementary material for: Maternal migraine and offspring ADHD: triangulating the evidence
Source: BMC Med. 2026 Feb 14;24:167. doi: 10.1186/s12916-026-04692-4 (PMC13011382; doi:10.1186/s12916-026-04692-4)
Supplement: Supplementary file 1 — Additional file 1. Notes 1–3. Note 1 Baseline characteristic assessment in Avon Longitudinal Study of Parents and Child. Note 2 Details on genotyping in Avon Longitudinal Study of Parents and Child. Note 3 Mendelian randomizationmethod. [file 12916_2026_4692_MOESM1_ESM.docx]

**.Additional File 1**

[Supplement 1: Baseline Characteristic Assessment in ALSPAC 2](#_Toc203671157)

[Supplement 2: Details on genotyping in ALSPAC 4](#_Toc203671158)

[Supplement 3: Mendelian randomization method 4](#_Toc203671159)

# Note 1: Baseline Characteristic Assessment in ALSPAC

**Covariates (maternal characteristics):**

**Maternal Age when Pregnant**

Justification for inclusion: Evidence has shown that parental age may be linked to children's health outcomes. Maternal age has been associated to several offspring outcomes, including phenotypes related to neurodevelopmental and psychiatric conditions (autism spectrum disorder, bipolar disorder) [1]. The clinical presentation of migraine indicates that its prevalence reaches its peak between the ages of 20 and 40, while headache intensity tends to decrease [2].

In ALSPAC, maternal age at delivery was collected when the mother completed the ‘ALSPAC pregnancy, Birth and Infancy (pregnancy to 12 months)’ questionnaire and treated as a continuous variable.

**Maternal Alcohol Consumption during the First Trimester**

Justification for inclusion: A systemic review indicated that alcohol may trigger migraines, including the role of biogenic amines, sulfites, flavonoid phenols, serotonin release, and vasodilation effects [3]. A study based on MoBa found a weak but potentially causal link was observed concerning ADHD-related behaviours, suggesting that while prenatal alcohol exposure may not directly lead to an ADHD diagnosis, it could influence attention-related behaviours in offspring [4].

In ALSPAC, mothers were asked ‘How often have you drunk alcoholic drinks during the 1^st^ 3 months of this pregnancy?’ with options ‘Never’, ‘Less than 1 glass a week’, ‘At least 1 glass a week’, ‘1-2 glasses every day’, ‘At least 3-9 glasses every day’, and ‘At least 10 glasses every day’. Each glass means a pub measure of spirit, half of lager or cider, a wine glass of wine, etc. This variable was recategorized into ‘Never’, ‘Less than 1glass per week’, and ‘One or more than one glass per week’.

**Edinburgh Post-natal Depression Score (EPDS)**

Justification for inclusion: Migraine and depression have a well-established bidirectional relationship, with evidence suggesting shared genetic, neurobiological, and environmental factors contributing to their co-occurrence [5]. Maternal depression has been linked to a number of neurodevelopmental outcomes in the offspring [6].

In ALSPAC, maternal EPDS was collected from the questionnaire ‘ALSPAC pregnancy, Birth and Infancy (pregnancy to 12 months)’. The EDPS was developed to identify women who may have postpartum depression. Each answer is given a score of 0 to 3. The maximum score is 30. A score of more than 10 suggests minor or major depression may be present.

**Total Crown Crisp Experimental Index (CCEI)**

Justification for inclusion: Longitudinal studies have indicated that neurotic traits may predict the progression from episodic to chronic migraine. For instance, research has shown that individuals with higher levels of psychological distress and neuroticism are more susceptible to developing chronic headaches. Additionally, increased emotional reactivity and inadequate stress management have been associated with a higher frequency and intensity of migraine attacks [7, 8]. For offspring ADHD, some mothers of children with attention-deficit/hyperactivity disorder (ADHD) exhibit maladaptive personality traits, such as high neuroticism and low conscientiousness. Research has shown that children of mothers with these traits may respond less effectively to ADHD treatment. This suggests that maternal personality characteristics could influence the treatment outcomes of children with ADHD[9].

In ALSPAC, Maternal total Crown Crisp score was collected from the questionnaire ‘ALSPAC pregnancy, Birth and Infancy (pregnancy to 12 months)’. The Crown-Crisp Experiential Index (CCEI) was designed as a measure of neurotic symptomatology across the different subscales measured by the instrument, including anxiety, depression, and somaticism. Scores on the 6 scales of the CCEI were summed to derive the total score.

**Covariates (Socioeconomic status):**

Justification for inclusion: For migraine, a study found that individuals with lower educational levels had an increased likelihood of experiencing migraines [10]. Research also indicates that individuals from lower SES backgrounds have a higher prevalence of migraines [11]. For offspring ADHD, research consistently indicates a strong relationship between family socioeconomic status (SES) and the risk of childhood ADHD [12]. Studies have found that children of single mothers may have a higher risk of ADHD compared to those from two-parent households [13]. A study analysing data from birth cohorts across six countries found that children from families with lower household income or maternal education levels were more likely to have ADHD at ages 9-11[14].

**Maternal Highest Educational Attainment**

In ALSPAC, maternal highest education qualification was collected by questionnaires at 32 weeks’ gestation. Education level was first classified to ‘CSE’, ‘Vocational’, ‘O level’, ‘A level’, and ‘Degree’. When running the regression model, ‘CSE’ and ‘Vocational’ were combined as ‘CSE/Vocational’. Participants who did not reply the questionnaire were classified as ‘missing’ for this variable.

**Maternal Social Class**

In ALSPAC, maternal social class was assessed through maternal questionnaires that collected occupational data at 32 weeks gestation. For more detail on this occupational social class, see: https://sru.soc.surrey.ac.uk/SRU9.html.

**Maternal Marital Status**

In ALSPAC, mothers were asked about their marital status by questionnaires at 8 weeks’ gestation. Mothers were asked ‘What is your present marital status’ with options ‘never married’, ‘widowed’, ‘divorced’, ‘separated’, ‘married (once only)’, ‘married for second or third time’. This variable was recategorized as ‘Never’, ‘Married (once or more than once)’, and ‘Other’ due to the small sample size.

# Note 2: Details on genotyping in ALSPAC

A total of 10 015 women (mothers from the ALSPAC cohort) were genotyped using the Illumina 660 quad SNP chip which contains 557 124 SNP markers. Markers with minor allele frequency < 1%, SNPs with >5% missing genotypes and any markers that failed an exact test of Hardy-Weinberg equilibrium (P < 1 x 10^-6^) were excluded from further analyses. Genome-wide identity by state sharing was calculated for each pair of individuals in the cohort to identify cryptic relatedness.

In order to identify individuals who might have ancestries other than Western European, we merged data from both cohorts with the 60 western European (CEU) founder, 60 Nigerian (YRI) founder and 90 Japanese (JPT) and Han Chinese (CHB) individuals from the International HapMap Project. Genome-wide IBS distances for each pair of individuals were calculated on markers shared between the HapMap and the Illumina 660K SNP chip, and then the multidimensional scaling option in R was used to generate a two-dimensional plot based upon individuals' scores on the first two principal coordinates from this analysis. Samples that did not cluster with the CEU individuals were excluded from subsequent analyses. In addition, we plotted the proportion of missing data for each individual against their genome-wide heterozygosity. Any individual, who did not cluster with others, was removed from further analyses. Samples were excluded if they displayed more than 5% missingness, had indeterminate X chromosome heterozygosity or extreme autosomal heterozygosity. Samples showing evidence of population stratification were identified by multidimensional scaling of genome-wide identity by state pairwise distances using the four HapMap populations as a reference and then excluded. Cryptic relatedness was assessed using a IBD estimate of more than 0.125 which is expected to correspond to roughly 12.5% alleles shared IBD or a relatedness at the first cousin level. Related subjects that passed all other quality control thresholds were retained during subsequent phasing and imputation. 9,048 subjects and 526,688 SNPs passed these quality control filters.

A total of 477,482 SNPs shared between maternal and child samples were jointly analyzed. Additional genotypes were imputed using IMPUTE version2 to a 1000 Genomes reference panel that contained all available ethnicities with singleton and monomorphic sites removed. SNPs with >1% missingness (11,396 removed) and 321 subjects with potential ID mismatches were excluded. This yielded 17,842 individuals (6,305 duos) and 465,740 SNPs (112 removed during liftover, 234 failing HWE). Haplotypes were phased using ShapeIT (v2.r644), incorporating relatedness, and imputed against the 1000 Genomes Phase 1 (v3) reference panel using Impute V2.2.2, leveraging all 2,186 reference haplotypes, including non-European variants. This gave 8,237 eligible children and 8,196 eligible mothers with available genotype data after exclusion of related subjects, more details can be found at: <https://proposals.epi.bristol.ac.uk/alspac_omics_data_catalogue.html#orgb745137>

# Note 3: Mendelian randomization method

Inverse Variance Weighted (IVW): IVW combines the individual SNP-exposure and SNP-outcome associations using a weighted linear regression approach, where the weights are proportional to the inverse of the variance of the SNP-exposure associations [15]. This method assumes that all genetic variants are valid instrumental variables and that there is no horizontal pleiotropy (i.e., genetic variants affect the outcome only through the exposure of interest).

MR-Egger: MR-Egger does not assume that all genetic variants used as instrumental variables are valid [16]. Instead, it allows for the possibility of horizontal pleiotropy by estimating the intercept term, which represents the average pleiotropic effect across all genetic variants. This intercept term is then used to adjust the causal estimate for the presence of pleiotropy. MR-Egger regression provides unbiased estimates of causal effects even in the presence of horizontal pleiotropy, as long as certain assumptions are met. However, it typically requires larger sample sizes to achieve sufficient statistical power compared to other MR methods like IVW.

The Weighted Median method: a robust approach used to estimate causal effects when there are heterogeneity and potential horizontal pleiotropy among genetic variants used as instrumental variables [17]. The Weighted Median method provides a consistent estimate of the causal effect under certain assumptions, even if up to 50% of the weight in the analysis comes from invalid instruments. It is considered a valuable tool in MR analysis, particularly when there is uncertainty about the presence of horizontal pleiotropy or when there is heterogeneity among genetic instruments.

Weighted Mode method: Similar to the Weighted Median method, the Weighted Mode method provides a consistent estimate of the causal effect under certain assumptions, even when a substantial proportion of the genetic instruments may be invalid [18]. It is useful in MR analysis when there is heterogeneity among genetic instruments and uncertainty about the presence of horizontal pleiotropy.

Reference

1. Bergh C, Pinborg A, Wennerholm U-B. Parental age and child outcomes. Fertil Steril. 2019;111:1036–1046.

2. Fila M, Pawlowska E, Szczepanska J, Blasiak J. Different Aspects of Aging in Migraine. Aging Dis. 2023;14:2028–2050.

3. Panconesi A. Alcohol and migraine: trigger factor, consumption, mechanisms. A review. J Headache Pain. 2008;9:19–27.

4. Eilertsen EM, Gjerde LC, Reichborn-Kjennerud T, Ørstavik RE, Knudsen GP, Stoltenberg C, et al. Maternal alcohol use during pregnancy and offspring attention-deficit hyperactivity disorder (ADHD): a prospective sibling control study. Int J Epidemiol. 2017;46:1633–1640.

5. Jahangir S, Adjepong D, Al-Shami HA, Malik BH. Is There an Association Between Migraine and Major Depressive Disorder? A Narrative Review. Cureus. 2020. 10 June 2020. https://doi.org/10.7759/cureus.8551.

6. Nidey NL, Momany AM, Strathearn L, Carter KD, Wehby GL, Bao W, et al. Association between perinatal depression and risk of attention deficit hyperactivity disorder among children: a retrospective cohort study. Ann Epidemiol. 2021;63:1–6.

7. Radat F, Swendsen J. Psychiatric Comorbidity in Migraine: A Review. Cephalalgia. 2005;25:165–178.

8. Ashina S, Lyngberg A, Jensen R. Headache characteristics and chronification of migraine and                    tension-type headache: A population-based study. Cephalalgia. 2010;30:943–954.

9. Perez Algorta G, MacPherson HA, Arnold LE, Hinshaw SP, Hechtman L, Sibley MH, et al. Maternal personality traits moderate treatment response in the Multimodal Treatment Study of attention-deficit/hyperactivity disorder. Eur Child Adolesc Psychiatry. 2020;29:1513–1524.

10. Winter AC, Berger K, Buring JE, Kurth T. Associations of socioeconomic status with migraine and non-migraine headache. Cephalalgia. 2011;32:159–170.

11. Stewart WF, Roy J, Lipton RB. Migraine prevalence, socioeconomic status, and social causation. Neurology. 2013;81:948–955.

12. Russell AE, Ford T, Russell G. Socioeconomic Associations with ADHD: Findings from a Mediation Analysis. PLoS One. 2015;10:e0128248-.

13. Schermerhorn AC, DOnofrio BM, Slutske WS, Emery RE, Turkheimer E, Harden KP, et al. Offspring ADHD as a risk factor for parental marital problems: Controls for genetic and environmental confounds. Twin Research and Human Genetics. 2012;15:700–713.

14. Spencer NJ, Ludvigsson J, Bai G, Gauvin L, Clifford SA, Abu Awad Y, et al. Social gradients in ADHD by household income and maternal education exposure during early childhood: Findings from birth cohort studies across six countries. PLoS One. 2022;17:e0264709-.

15. Davey Smith G, Hemani G. Mendelian randomization: genetic anchors for causal inference in epidemiological studies. Hum Mol Genet. 2014;23:R89–R98.

16. Burgess S, Thompson SG. Interpreting findings from Mendelian randomization using the MR-Egger method. Eur J Epidemiol. 2017;32:377–389.

17. Bowden J, Davey Smith G, Haycock PC, Burgess S. Consistent Estimation in Mendelian Randomization with Some Invalid Instruments Using a Weighted Median Estimator. Genet Epidemiol. 2016;40:304–314.

18. Hartwig FP, Davey Smith G, Bowden J. Robust inference in summary data Mendelian randomization via the zero modal pleiotropy assumption. Int J Epidemiol. 2017;46:1985–1998.
