# Supplementary material for: Maternal migraine and offspring ADHD: triangulating the evidence
Source: BMC Med. 2026 Feb 14;24:167. doi: 10.1186/s12916-026-04692-4 (PMC13011382; doi:10.1186/s12916-026-04692-4)
Supplement: Supplementary file 2 — Additional file 2. Tables S1–S10. Table S1 The baseline characteristics for mothers who completed and did not complete questionnaires collected at 12 weeks’ gestation. Table S2 Associations between polygenic risk scoresand migraine/ADHD traits. Table S2A Association between maternal migraine PRS and offspring ADHD traits. Table S2B Association between ADHD-PRS and migrainein mothers from ALSPAC. Table S3 Harmonized SNP data for two-sample MR to estimate the causal effect of genetic liability to migraine on ADHD. Table S4 Heterogeneity and horizontal pleiotropy test for IVs. Table S5 Estimated causal effect of genetic liability to migraine on ADHD using IVs validated in ALSPAC data during pregnancy. Table S6 Harmonized SNP data for two-sample MR to estimate the causal effect of genetic liability to migraine on ADHD. Table S7 Harmonized SNP data for two-sample MR to estimate the causal effect of genetic liability of migraine on ADHD. Table S8 Secondary sensitivity analysis—MR estimation for causal effect of genetic liability to migraine on ADHD. Table S9 Harmonized SNP data for two-sample MR to estimate the causal effect of genetic liability to ADHD on migraine. Table S10 Heterogeneity and horizontal pleiotropy test for IVs. [file 12916_2026_4692_MOESM2_ESM.docx]

Additional File 2

[Table S1 1](#_Toc203671066)

[Table S2 3](#_Toc203671067)

[Table S3 4](#_Toc203671068)

[Table S4 6](#_Toc203671069)

[Table S5 7](#_Toc203671070)

[Table S6 8](#_Toc203671071)

[Table S7 13](#_Toc203671072)

[Table S8 14](#_Toc203671073)

[Table S9 15](#_Toc203671074)

[Table S10 17](#_Toc203671075)

Table S1. The baseline characteristics for mothers who completed and did not complete questionnaires collected at 12 week's gestation.

| Variable | Did not complete  (N=3346) | Completed  (N=12 299) | All participants  (N=15 645) | P value |
| --- | --- | --- | --- | --- |
|  | Number (%) | Number (%) | Number (%) |  |
| ***Total*** | 3346 (100) | 12 299 (%) | 15 645 (%) |  |
| ***Maternal Highest Education Attainment*** |  |  |  | 5.23×10^-35^ |
| A level | 130 (14.5) | 2662 (23) | 2792 (22.4) |  |
| CSE/Vocational | 434 (48.4) | 3319 (28.7) | 3753 (30.1) |  |
| Degree | 69 (7.7) | 1537 (13.3) | 1606 (12.9) |  |
| O level | 263 (29.4) | 4055 (35) | 4318 (34.6) |  |
| Missing | 2450 | 726 | 3176 |  |
| ***Age at pregnancy*** |  |  |  | 3.23×10^-24^ |
| Mean (SD) | 26.6 (5.4) | 27.9 (4.8) | 27.8 (4.9) |  |
| Missing | 1801 | 350 | 2151 |  |
| ***Maternal social class*** |  |  |  | 2.18×10^-10^ |
| I | 29 (5.3) | 566 (5.9) | 595 (5.9) |  |
| II | 140 (25.5) | 3038 (31.8) | 3178 (31.5) |  |
| III (non-manual) | 207 (37.7) | 4112 (43.1) | 4319 (42.8) |  |
| III (manual) | 62 (11.3) | 728 (7.6) | 790 (7.8) |  |
| IV | 86 (15.7) | 909 (9.5) | 995 (9.9) |  |
| V | 25 (4.6) | 197 (2.1) | 222 (2.2) |  |
| Missing | 2797 | 2749 | 5546 |  |
| ***Marital Status*** |  |  |  | 1.29×10^-35^ |
| 1st marriage | 855 (55.3) | 8388 (70) | 9243 (68.3) |  |
| marriage 2 or 3 | 101 (6.5) | 780 (6.5) | 881 (6.5) |  |
| never | 469 (30.4) | 2126 (17.7) | 2595 (19.2) |  |
| widowed/divorced/separated | 120 (7.8) | 696 (5.8) | 816 (6) |  |
| Missing | 1801 | 309 | 2110 |  |
| ***Alcohol consumption during 1 to 3 months pregnancy*** | |  |  |  |
| Never | 603 (48.1) | 5385 (45.2) | 5988 (45.5) | 5×10^-4^ |
| <1 glass per week | 424 (33.8) | 4672 (39.2) | 5096 (38.7) |  |
| 1+ glass per week | 227 (18.1) | 1860 (15.6) | 2087 (15.8) |  |
| Missing | 2092 | 382 | 2474 |  |
| ***EPDS*** |  |  |  | 1.25×10^-28^ |
| Mean (SD) | 9 (5.2) | 7.3 (4.6) | 7.4 (4.7) |  |
| Missing | 2325 | 1926 | 4251 |  |
| ***CCEI*** |  |  |  | 6.95×10^-16^ |
| Mean (SD) | 15.6(8.5) | 13.6(7.7) | 13.7(7.8) |  |
| Missing | 2365 | 1763 | 4128 |  |
| ***Offspring ADHD symptoms at age 7*** |  |  |  | 3.71×10^-3^ |
| No | 299 (84.2) | 7170 (89.3) | 7469 (89.1) |  |
| Yes | 56 (15.8) | 861 (10.7) | 917 (10.9) |  |
| Missing | 2991 | 4268 | 7259 |  |

BMI: body mass index; SD: standard deviation; EPDS: Edinburgh Postnatal Depression Score; CCEI: Crown-Crisp Experimental Index.

For category variables, the number in the cells indicate the number of participants and corresponding percentage/distribution (number in brackets).

Table S2. Associations between polygenic risk scores (PRS) and migraine/ADHD traits.

Table S2A. Association between maternal migraine PRS and offspring ADHD traits.

| Variable ^1^ | No.SNP | R^2^ (CoxSnell) ^2^ | OR (95% CI) | P value |
| --- | --- | --- | --- | --- |
| PRS.1 (P < 5×10^-8^) | 40 | 0.008365311 | 1.04 (0.95, 1.14) | 0.396 |
| PRS.2 (P < 5×10^-6^) | 138 | 0.016617017 | 1.03 (0.95, 1.13) | 0.529 |
| PRS.3 (P < 1×10^-5^) | 191 | 0.020652737 | 1 (0.91, 1.09) | 0.913 |
| PRS.4 (P < 0.001) | 1976 | 0.086207971 | 1.08 (0.99, 1.18) | 0.097 |
| PRS.5 (P < 0.005) | 5424 | 0.140577702 | 1.14 (1.04, 1.25) | 4.17×10^-3^ |
| PRS.6 (P < 0.05) | 20561 | 0.245205646 | 1.17 (1.07, 1.28) | 5.72×10^-4^ |
| PRS.7 (P < 0.1) | 29880 | 0.260126967 | 1.19 (1.09, 1.3) | 1.02×10^-4^ |
| PRS.8 (P < 0.5) | 64620 | 0.299024704 | 1.21 (1.11, 1.32) | 3.33×10^-5^ |

^1^ PRS thresholds indicate the p-value cut-offs used for SNP inclusion.

^2^ R² values are reported as fractions ranging from 0 to 1. Because ALSPAC participants contributed to the discovery GWAS for migraine, prediction accuracy of the PRS within ALSPAC is likely to be inflated due to sample overlap. Accordingly, the pseudo-R² values reported here should be interpreted with caution and viewed primarily as evidence of directionality rather than absolute predictive power.

Models were adjusted for offspring's sex, and first 10 principal components.

Table S2B. Association between ADHD PRS and migraine (ever vs. never) in mothers from ALSPAC (Ncontrol = 3933; Ncase = 2989).

| Variable | No.SNP | OR (95% CI) | P value |
| --- | --- | --- | --- |
| PRS.1 (P < 5×10^-8^) | 26 | 1.01 (0.96, 1.06) | 0.687 |
| PRS.2 (P < 5×10^-6^) | 155 | 1.04 (0.99, 1.09) | 0.111 |
| PRS.3 (P < 1×10^-5^) | 206 | 1.06 (1.01, 1.11) | 0.018 |
| PRS.4 (P < 0.001) | 2138 | 1.07 (1.02, 1.12) | 0.005 |
| PRS.5 (P < 0.005) | 5229 | 1.09 (1.04, 1.14) | 4.5×10^-4^ |
| PRS.6 (P < 0.05) | 18133 | 1.11 (1.06, 1.16) | 3.28×10^-5^ |
| PRS.7 (P < 0.1) | 26119 | 1.13 (1.07, 1.18) | 1.62×10^-6^ |
| PRS.8 (P < 0.5) | 55521 | 1.11 (1.06, 1.17) | 1.06×10^-5^ |

PRS thresholds indicate the p-value cut-offs used for SNP inclusion.

Models were adjusted for participants’ first 10 principal components.

Table S3. Harmonized SNP data for two-sample MR to estimate the causal effect of genetic liability to migraine on ADHD.

| SNP | A1 | A2 | Beta. exposure | SE. exposure | P. exposure | Beta. outcome | SE. outcome | P. outcome | EAF. exposure | EAF. outcome | Direction with ALSPAC | F |
| --- | --- | --- | --- | --- | --- | --- | --- | --- | --- | --- | --- | --- |
| rs10166942 | C | T | -0.1200 | 0.0097 | 5.89×10^-32^ | 0.0123 | 0.0116 | 0.29 | 0.20 | 0.18 | FALSE | 138.59 |
| rs1019990 | T | C | -0.0554 | 0.0084 | 3.87×10^-11^ | 0.0135 | 0.0099 | 0.18 | 0.30 | 0.28 | TRUE | 43.75 |
| rs10234636 | T | C | 0.1100 | 0.0117 | 6.6×10^-21^ | -0.0002 | 0.0151 | 0.99 | 0.11 | 0.10 | FALSE | 88.10 |
| rs1025497 | A | G | -0.0499 | 0.0079 | 2.89×10^-10^ | 0.0001 | 0.0097 | 1.00 | 0.37 | 0.39 | TRUE | 39.81 |
| rs10456100 | T | C | 0.0607 | 0.0085 | 7.75×10^-13^ | 0.0186 | 0.0106 | 0.08 | 0.28 | 0.26 | FALSE | 51.43 |
| rs10833535 | A | G | 0.0530 | 0.0077 | 6.89×10^-12^ | -0.0116 | 0.0094 | 0.22 | 0.48 | 0.48 | FALSE | 47.13 |
| rs10849061 | C | T | 0.0632 | 0.0076 | 9.36×10^-17^ | -0.0077 | 0.0093 | 0.41 | 0.49 | 0.47 | FALSE | 69.20 |
| rs11153082 | G | A | 0.0977 | 0.0080 | 6.02×10^-34^ | -0.0060 | 0.0099 | 0.54 | 0.32 | 0.34 | TRUE | 147.70 |
| rs11172113 | C | T | -0.1170 | 0.0078 | 1.15×10^-51^ | -0.0005 | 0.0096 | 0.96 | 0.42 | 0.39 | TRUE | 228.93 |
| rs112255710 | T | C | -0.0994 | 0.0146 | 1.13×10^-11^ | 0.0039 | 0.0183 | 0.83 | 0.08 | 0.09 | TRUE | 46.16 |
| rs11624776 | C | A | -0.0516 | 0.0084 | 8.34×10^-10^ | 0.0176 | 0.0102 | 0.08 | 0.31 | 0.33 | TRUE | 37.74 |
| rs11782673 | G | A | -0.0570 | 0.0103 | 3.46×10^-8^ | 0.0044 | 0.0128 | 0.73 | 0.16 | 0.17 | TRUE | 30.48 |
| rs12025158 | A | G | 0.0509 | 0.0080 | 1.69×10^-10^ | -0.0044 | 0.0098 | 0.65 | 0.35 | 0.38 | FALSE | 40.86 |
| rs12260436 | C | A | 0.0535 | 0.0087 | 6.55×10^-10^ | -0.0205 | 0.0121 | 0.09 | 0.25 | 0.27 | FALSE | 38.21 |
| rs13078967 | C | A | -0.1630 | 0.0252 | 1.11×10^-10^ | 0.0424 | 0.0344 | 0.22 | 0.03 | 0.03 | FALSE | 41.69 |
| rs17303101 | A | G | 0.0691 | 0.0084 | 2.37×10^-16^ | 0.0236 | 0.0105 | 0.02 | 0.29 | 0.30 | TRUE | 67.37 |
| rs1925950 | G | A | 0.0760 | 0.0079 | 8.49×10^-22^ | -0.0003 | 0.0098 | 0.97 | 0.35 | 0.33 | FALSE | 92.16 |
| rs2078371 | C | T | 0.1310 | 0.0115 | 5.34×10^-30^ | -0.0233 | 0.0147 | 0.11 | 0.12 | 0.11 | FALSE | 129.64 |
| rs2274224 | C | G | -0.0659 | 0.0077 | 1.34×10^-17^ | -0.0059 | 0.0089 | 0.51 | 0.43 | 0.44 | TRUE | 73.03 |
| rs2672592 | T | G | 0.0441 | 0.0079 | 1.97×10^-8^ | -0.0065 | 0.0098 | 0.51 | 0.36 | 0.36 | FALSE | 31.57 |
| rs28451064 | A | G | -0.0660 | 0.0118 | 2.26×10^-8^ | 0.0253 | 0.0146 | 0.08 | 0.13 | 0.13 | FALSE | 31.31 |
| rs34273564 | T | C | 0.0423 | 0.0076 | 3.06×10^-8^ | -0.0013 | 0.0094 | 0.89 | 0.48 | 0.47 | FALSE | 30.72 |
| rs42854 | G | C | 0.0645 | 0.0081 | 1.63×10^-15^ | -0.0111 | 0.0101 | 0.27 | 0.31 | 0.32 | FALSE | 63.57 |
| rs4910165 | C | G | -0.0664 | 0.0082 | 4.23×10^-16^ | 0.0027 | 0.0095 | 0.78 | 0.32 | 0.31 | FALSE | 66.23 |
| rs6046147 | T | C | 0.0627 | 0.0087 | 5.61×10^-13^ | -0.0063 | 0.0106 | 0.55 | 0.25 | 0.26 | FALSE | 52.06 |
| rs6057599 | T | C | 0.0447 | 0.0081 | 3.20×10^-8^ | 0.0281 | 0.0098 | 0.00 | 0.34 | 0.31 | FALSE | 30.63 |
| rs6904682 | T | C | -0.0451 | 0.0076 | 3.52×10^-9^ | -0.0145 | 0.0095 | 0.13 | 0.45 | 0.44 | TRUE | 34.93 |
| rs72926788 | C | T | -0.1300 | 0.0232 | 2.02×10^-8^ | -0.0669 | 0.0245 | 0.01 | 0.04 | 0.04 | TRUE | 31.52 |
| rs7518255 | A | G | 0.1220 | 0.0090 | 1.66×10^-41^ | -0.0367 | 0.0136 | 0.01 | 0.22 | 0.25 | FALSE | 182.31 |
| rs7544256 | G | A | -0.0509 | 0.0080 | 1.67×10^-10^ | -0.0001 | 0.0099 | 0.99 | 0.35 | 0.36 | TRUE | 40.88 |
| rs7640543 | A | G | 0.0461 | 0.0081 | 1.34×10^-8^ | -0.0199 | 0.0100 | 0.05 | 0.32 | 0.33 | FALSE | 32.33 |
| rs7684253 | C | T | -0.0439 | 0.0077 | 1.00×10^-8^ | -0.0131 | 0.0094 | 0.16 | 0.45 | 0.45 | TRUE | 32.88 |
| rs7757975 | T | G | 0.0895 | 0.0104 | 6.47×10^-18^ | -0.0005 | 0.0126 | 0.97 | 0.16 | 0.14 | FALSE | 74.48 |
| rs8075138 | T | C | 0.0479 | 0.0078 | 9.07×10^-10^ | 0.0142 | 0.0097 | 0.15 | 0.40 | 0.38 | FALSE | 37.57 |
| rs9349379 | G | A | -0.0838 | 0.0080 | 6.69×10^-26^ | 0.0021 | 0.0098 | 0.83 | 0.41 | 0.40 | TRUE | 110.9 |
| rs950570 | T | C | 0.0847 | 0.0149 | 1.29×10^-8^ | 0.0289 | 0.0186 | 0.12 | 0.07 | 0.06 | FALSE | 32.39 |
| rs953588 | T | C | 0.0535 | 0.0078 | 7.38×10^-12^ | 0.0041 | 0.0098 | 0.68 | 0.38 | 0.36 | FALSE | 47.00 |

A1: effect allele; A2: reference allele

Table S4. Heterogeneity and horizontal pleiotropy test for IVs (migraine).

| exposure | outcome | method | Q statistic | Q-P value | beta for intercept | se for intercept | P for intercept |
| --- | --- | --- | --- | --- | --- | --- | --- |
| migraine | ADHD | Inverse variance weighted | 63.06 | 0.0025 |  |  |  |
| migraine | ADHD | MR Egger | 61.62 | 0.0026 | 0.0063 | 0.0071 | 0.379 |

Table S5. Estimated causal effect of genetic liability to migraine on ADHD using IVs validated in ALSPAC data during pregnancy.

| exposure | outcome | method | No. SNP | OR (95% CI) | P value | Q statistic | Q-P value |
| --- | --- | --- | --- | --- | --- | --- | --- |
| Migraine | ADHD | Inverse variance weighted ^1^ | 13 | 1.03 (0.92, 1.15) | 0.608 |  |  |
| Migraine | ADHD | Inverse variance weighted ^2^ | 13 | 1.03 (0.95, 1.11) | 0.49 | 21.72 | 0.041 |
| Migraine | ADHD | MR Egger | 13 | 1.04 (0.75, 1.44) | 0.834 | 21.72 | 0.027 |
| Migraine | ADHD | Weighted median | 13 | 1 (0.89, 1.12) | 0.98 |  |  |
| Migraine | ADHD | Weighted mode | 13 | 0.98 (0.86, 1.11) | 0.739 |  |  |
| Migraine | ADHD | Simple mode | 13 | 0.97 (0.81, 1.16) | 0.773 |  |  |

^1^ random effect Inverse variance weighed model

^2^ fixed effect Inverse variance model.

Table S6. Harmonized SNP data for two-sample MR to estimate the causal effect of genetic liability to migraine on ADHD (primary GWAS combined with 23andMe ).

| SNP | A1 | A2 | Beta. exposure | Beta. outcome | EAF. exposure | EAF. outcome | SE. outcome | P. outcome | SE. exposure | P. exposure | F |
| --- | --- | --- | --- | --- | --- | --- | --- | --- | --- | --- | --- |
| rs1003194 | A | G | 0.0343 | 0.0171 | 0.38 | 0.37 | 0.0098 | 0.080 | 0.0054 | 2.43×10^-10^ | 40.16 |
| rs10038882 | C | T | -0.0425 | 0.0037 | 0.25 | 0.26 | 0.0109 | 0.731 | 0.0060 | 1.33×10^-12^ | 50.36 |
| rs10128028 | C | T | -0.0298 | 0.0229 | 0.48 | 0.48 | 0.0093 | 0.014 | 0.0052 | 7.66×10^-9^ | 33.41 |
| rs10166942 | C | T | -0.0992 | 0.0123 | 0.20 | 0.18 | 0.0116 | 0.291 | 0.0066 | 3.34×10^-12^ | 224.76 |
| rs1019990 | T | C | -0.0387 | 0.0135 | 0.30 | 0.28 | 0.0099 | 0.175 | 0.0057 | 9.35×10^-51^ | 46.39 |
| rs10218452 | G | A | 0.1100 | -0.0337 | 0.23 | 0.26 | 0.0132 | 0.011 | 0.0062 | 1×10^-11^ | 317.06 |
| rs10234636 | T | C | 0.0889 | -0.0002 | 0.11 | 0.10 | 0.0151 | 0.988 | 0.0081 | 7.26×10^-71^ | 120.87 |
| rs10405121 | A | G | -0.0332 | 0.0034 | 0.45 | 0.46 | 0.0097 | 0.725 | 0.0053 | 4.43×10^-28^ | 38.84 |
| rs10456100 | T | C | 0.0507 | 0.0186 | 0.28 | 0.26 | 0.0106 | 0.081 | 0.0057 | 4.74×10^-10^ | 78.36 |
| rs10777902 | A | C | 0.0331 | -0.0019 | 0.50 | 0.50 | 0.0092 | 0.838 | 0.0051 | 9.16×10^-19^ | 41.44 |
| rs10828247 | A | G | -0.0340 | -0.0194 | 0.65 | 0.64 | 0.0099 | 0.049 | 0.0059 | 1.25×10^-10^ | 33.45 |
| rs10894756 | A | G | -0.0295 | -0.0305 | 0.43 | 0.45 | 0.0094 | 0.001 | 0.0053 | 7.51×10^-9^ | 30.87 |
| rs11031122 | C | T | 0.0366 | 0.0282 | 0.24 | 0.25 | 0.0109 | 0.010 | 0.0059 | 2.83×10^-8^ | 38.10 |
| rs11102915 | C | T | -0.0436 | -0.0004 | 0.36 | 0.36 | 0.0100 | 0.969 | 0.0054 | 6.91×10^-10^ | 66.28 |
| rs11153082 | G | A | 0.0840 | -0.0060 | 0.33 | 0.34 | 0.0099 | 0.541 | 0.0054 | 4.1×10^-16^ | 239.00 |
| rs11165300 | G | T | 0.0329 | -0.0140 | 0.24 | 0.24 | 0.0106 | 0.185 | 0.0060 | 7.26×10^-54^ | 29.88 |
| rs11172113 | C | T | -0.1070 | -0.0005 | 0.42 | 0.39 | 0.0096 | 0.961 | 0.0053 | 4.72×10^-8^ | 407.55 |
| rs11248546 | T | C | -0.0368 | 0.0060 | 0.44 | 0.45 | 0.0093 | 0.523 | 0.0052 | 1.38×10^-90^ | 50.01 |
| rs11624776 | C | A | -0.0498 | 0.0176 | 0.32 | 0.33 | 0.0102 | 0.084 | 0.0056 | 1.59×10^-12^ | 78.23 |
| rs11782789 | T | A | -0.0414 | 0.0044 | 0.16 | 0.17 | 0.0128 | 0.730 | 0.0070 | 9.75×10^-19^ | 35.21 |
| rs11957829 | G | A | 0.0411 | 0.0117 | 0.17 | 0.19 | 0.0131 | 0.371 | 0.0068 | 1.07×10^-8^ | 36.50 |
| rs12057629 | C | T | 0.0400 | -0.0046 | 0.36 | 0.38 | 0.0098 | 0.636 | 0.0054 | 3.03×10^-9^ | 55.59 |
| rs12226331 | T | A | 0.0396 | -0.0029 | 0.35 | 0.37 | 0.0100 | 0.772 | 0.0054 | 1.58×10^-9^ | 54.17 |
| rs12260436 | C | A | 0.0360 | -0.0205 | 0.26 | 0.27 | 0.0121 | 0.089 | 0.0058 | 9.38×10^-14^ | 38.01 |
| rs12295710 | T | C | 0.0447 | -0.0098 | 0.47 | 0.49 | 0.0098 | 0.319 | 0.0055 | 1.92×10^-13^ | 67.01 |
| rs12419507 | A | G | -0.0314 | 0.0125 | 0.40 | 0.41 | 0.0094 | 0.181 | 0.0053 | 7.29×10^-10^ | 34.43 |
| rs12452590 | G | T | 0.0375 | -0.0068 | 0.38 | 0.37 | 0.0118 | 0.569 | 0.0059 | 2.86×10^-16^ | 40.49 |
| rs1245463 | A | G | 0.0396 | -0.0046 | 0.39 | 0.39 | 0.0099 | 0.638 | 0.0053 | 4.54×10^-9^ | 56.55 |
| rs12653216 | T | C | 0.0370 | -0.0137 | 0.21 | 0.21 | 0.0111 | 0.217 | 0.0064 | 2.03×10^-10^ | 33.31 |
| rs12708529 | G | A | -0.0358 | -0.0395 | 0.27 | 0.25 | 0.0104 | 0.000 | 0.0058 | 5.72×10^-14^ | 37.80 |
| rs12712881 | A | C | 0.0328 | -0.0013 | 0.44 | 0.43 | 0.0093 | 0.889 | 0.0052 | 8.08×10^-9^ | 39.44 |
| rs1271309 | A | G | -0.0403 | 0.0099 | 0.16 | 0.15 | 0.0128 | 0.440 | 0.0073 | 8.11×10^-10^ | 30.33 |
| rs13078967 | C | A | -0.1460 | 0.0424 | 0.03 | 0.03 | 0.0344 | 0.218 | 0.0178 | 3.5×10^-10^ | 67.54 |
| rs13235543 | T | C | -0.0581 | -0.0226 | 0.13 | 0.12 | 0.0141 | 0.109 | 0.0080 | 3.74×10^-8^ | 53.25 |
| rs138556413 | T | C | -0.1290 | -0.0688 | 0.04 | 0.04 | 0.0246 | 0.005 | 0.0159 | 6.85×10^-9^ | 66.26 |
| rs1458170 | T | C | -0.0416 | 0.0208 | 0.16 | 0.17 | 0.0126 | 0.099 | 0.0071 | 2.16×10^-16^ | 33.97 |
| rs1472662 | T | G | 0.0352 | 0.0068 | 0.22 | 0.21 | 0.0113 | 0.547 | 0.0062 | 3.06×10^-13^ | 31.79 |
| rs1499963 | T | C | -0.0321 | -0.0040 | 0.32 | 0.33 | 0.0099 | 0.688 | 0.0056 | 4.15×10^-16^ | 33.46 |
| rs1542668 | A | G | -0.0307 | -0.0125 | 0.33 | 0.35 | 0.0098 | 0.203 | 0.0055 | 5.75×10^-9^ | 31.09 |
| rs17723637 | G | A | 0.0415 | 0.0472 | 0.15 | 0.16 | 0.0127 | 0.000 | 0.0072 | 1.75×10^-8^ | 33.17 |
| rs1982072 | T | A | -0.0372 | 0.0013 | 0.30 | 0.30 | 0.0102 | 0.896 | 0.0056 | 7.48×10^-9^ | 43.58 |
| rs2000660 | A | G | 0.0496 | -0.0364 | 0.09 | 0.11 | 0.0220 | 0.098 | 0.0091 | 2.53×10^-8^ | 29.78 |
| rs2078371 | C | T | 0.1060 | -0.0233 | 0.12 | 0.11 | 0.0147 | 0.112 | 0.0078 | 8.63×10^-9^ | 184.41 |
| rs2119930 | G | T | 0.0409 | 0.0141 | 0.41 | 0.39 | 0.0101 | 0.162 | 0.0052 | 4.22×10^-11^ | 60.77 |
| rs2124663 | T | C | -0.0375 | -0.0021 | 0.22 | 0.23 | 0.0156 | 0.893 | 0.0063 | 4.95×10^-8^ | 35.64 |
| rs2160875 | C | T | 0.0649 | -0.0092 | 0.48 | 0.47 | 0.0093 | 0.320 | 0.0052 | 5.87×10^-42^ | 158.44 |
| rs2274319 | T | C | 0.0727 | 0.0001 | 0.35 | 0.33 | 0.0098 | 0.994 | 0.0054 | 6.69×10^-15^ | 181.35 |
| rs246326 | T | C | 0.0476 | 0.0092 | 0.13 | 0.13 | 0.0141 | 0.514 | 0.0077 | 2.44×10^-9^ | 38.14 |
| rs2483262 | T | G | 0.0483 | -0.0193 | 0.23 | 0.23 | 0.0115 | 0.095 | 0.0060 | 2.72×10^-36^ | 63.92 |
| rs2672592 | T | G | 0.0380 | -0.0065 | 0.36 | 0.36 | 0.0098 | 0.508 | 0.0054 | 3.28×10^-26^ | 50.53 |
| rs28451064 | A | G | -0.0633 | 0.0253 | 0.13 | 0.13 | 0.0146 | 0.083 | 0.0080 | 2.74×10^-41^ | 62.05 |
| rs28455731 | T | G | 0.0690 | 0.0007 | 0.16 | 0.14 | 0.0126 | 0.954 | 0.0070 | 6.8×10^-10^ | 96.65 |
| rs28739509 | C | T | 0.0386 | -0.0018 | 0.27 | 0.26 | 0.0111 | 0.871 | 0.0061 | 1.36×10^-15^ | 39.99 |
| rs28756401 | A | G | -0.0335 | 0.0319 | 0.29 | 0.28 | 0.0102 | 0.002 | 0.0058 | 1.22×10^-12^ | 33.77 |
| rs28929474 | T | C | 0.1110 | 0.0906 | 0.02 | 0.02 | 0.0336 | 0.007 | 0.0186 | 3.52×10^-15^ | 35.56 |
| rs3092262 | G | A | 0.0299 | 0.0056 | 0.46 | 0.47 | 0.0094 | 0.554 | 0.0052 | 8.82×10^-23^ | 32.74 |
| rs34273564 | T | C | 0.0336 | -0.0013 | 0.48 | 0.47 | 0.0094 | 0.891 | 0.0052 | 2.64×10^-10^ | 41.89 |
| rs34624768 | G | A | 0.0385 | -0.0016 | 0.40 | 0.42 | 0.0094 | 0.863 | 0.0052 | 6.4×10^-9^ | 54.41 |
| rs34914463 | C | T | -0.0492 | -0.0279 | 0.13 | 0.11 | 0.0150 | 0.063 | 0.0082 | 2.54×10^-9^ | 35.66 |
| rs3891689 | C | T | 0.0577 | 0.0113 | 0.23 | 0.24 | 0.0110 | 0.303 | 0.0061 | 1.08×10^-8^ | 90.22 |
| rs4278223 | A | T | -0.0451 | 0.0008 | 0.36 | 0.38 | 0.0129 | 0.952 | 0.0073 | 1×10^-10^ | 38.31 |
| rs42854 | G | C | 0.0394 | -0.0111 | 0.31 | 0.32 | 0.0101 | 0.269 | 0.0055 | 1.71×10^-13^ | 51.04 |
| rs4668251 | C | G | -0.0334 | 0.0015 | 0.29 | 0.29 | 0.0106 | 0.887 | 0.0058 | 2.41×10^-9^ | 33.43 |
| rs4705403 | A | G | 0.0471 | 0.0165 | 0.10 | 0.12 | 0.0163 | 0.312 | 0.0083 | 2.28×10^-21^ | 32.56 |
| rs4739105 | T | C | 0.0356 | 0.0091 | 0.21 | 0.23 | 0.0116 | 0.430 | 0.0064 | 6.24×10^-10^ | 30.86 |
| rs4814864 | C | G | 0.0648 | -0.0066 | 0.25 | 0.26 | 0.0107 | 0.536 | 0.0058 | 9.4×10^-13^ | 123.06 |
| rs4842676 | G | C | -0.0410 | 0.0346 | 0.18 | 0.16 | 0.0123 | 0.005 | 0.0069 | 7.58×10^-9^ | 35.79 |
| rs4907224 | T | A | -0.0358 | 0.0003 | 0.32 | 0.32 | 0.0099 | 0.976 | 0.0059 | 1.18×10^-8^ | 36.44 |
| rs4910165 | C | G | -0.0568 | 0.0027 | 0.32 | 0.31 | 0.0095 | 0.780 | 0.0055 | 2.85×10^-8^ | 105.35 |
| rs55707505 | C | T | -0.0307 | -0.0290 | 0.33 | 0.32 | 0.0100 | 0.004 | 0.0055 | 1.44×10^-28^ | 31.12 |
| rs56067931 | T | C | -0.0355 | -0.0035 | 0.20 | 0.20 | 0.0116 | 0.764 | 0.0065 | 2.26×10^-9^ | 29.83 |
| rs56140113 | T | C | -0.0368 | -0.0153 | 0.22 | 0.19 | 0.0112 | 0.173 | 0.0064 | 1.63×10^-9^ | 33.39 |
| rs566673 | G | T | 0.0302 | -0.0021 | 0.46 | 0.46 | 0.0093 | 0.822 | 0.0053 | 1.09×10^-24^ | 33.08 |
| rs580845 | C | A | -0.0299 | -0.0076 | 0.40 | 0.41 | 0.0101 | 0.451 | 0.0055 | 2.48×10^-8^ | 30.05 |
| rs6057599 | T | C | 0.0413 | 0.0281 | 0.34 | 0.31 | 0.0098 | 0.004 | 0.0055 | 4.83×10^-8^ | 55.72 |
| rs616060 | A | G | 0.0380 | -0.0065 | 0.36 | 0.36 | 0.0098 | 0.508 | 0.0054 | 7.76×10^-9^ | 39.15 |
| rs6556059 | T | C | 0.0336 | 0.0126 | 0.36 | 0.38 | 0.0100 | 0.207 | 0.0055 | 9.07×10^-9^ | 37.78 |
| rs6568677 | A | G | 0.0352 | 0.0344 | 0.21 | 0.22 | 0.0113 | 0.002 | 0.0063 | 4.3×10^-8^ | 31.46 |
| rs6668908 | T | G | -0.0307 | -0.0057 | 0.33 | 0.32 | 0.0100 | 0.571 | 0.0055 | 8.73×10^-14^ | 31.34 |
| rs6693567 | C | T | 0.0435 | -0.0015 | 0.27 | 0.25 | 0.0108 | 0.892 | 0.0059 | 4.04×10^-10^ | 55.01 |
| rs68002561 | G | A | 0.0591 | -0.0051 | 0.09 | 0.09 | 0.0168 | 0.761 | 0.0094 | 8.16×10^-10^ | 39.38 |
| rs7034179 | T | C | 0.0431 | 0.0012 | 0.43 | 0.41 | 0.0094 | 0.903 | 0.0052 | 2.09×10^-8^ | 68.14 |
| rs72923449 | C | A | 0.0777 | 0.0674 | 0.04 | 0.03 | 0.0259 | 0.009 | 0.0142 | 2.22×10^-8^ | 29.90 |
| rs73138150 | T | A | 0.0326 | -0.0489 | 0.32 | 0.32 | 0.0104 | 0.000 | 0.0058 | 1.25×10^-13^ | 31.59 |
| rs7335684 | G | A | 0.0342 | 0.0055 | 0.25 | 0.23 | 0.0106 | 0.608 | 0.0060 | 3.61×10^-10^ | 32.80 |
| rs73805934 | C | G | -0.0419 | -0.0033 | 0.18 | 0.17 | 0.0121 | 0.786 | 0.0069 | 1.6×10^-16^ | 37.18 |
| rs74182632 | A | G | 0.0638 | 0.0423 | 0.05 | 0.05 | 0.0188 | 0.024 | 0.0112 | 4.66×10^-8^ | 32.19 |
| rs74434374 | A | C | -0.0737 | 0.0420 | 0.05 | 0.05 | 0.0191 | 0.028 | 0.0126 | 1.95×10^-8^ | 34.44 |
| rs7511672 | A | G | -0.0313 | -0.0087 | 0.46 | 0.46 | 0.0091 | 0.341 | 0.0052 | 1.05×10^-8^ | 36.68 |
| rs7564469 | C | T | 0.0412 | 0.0045 | 0.16 | 0.16 | 0.0129 | 0.729 | 0.0071 | 1.11×10^-9^ | 34.22 |
| rs764508 | C | T | 0.0315 | -0.0048 | 0.37 | 0.36 | 0.0096 | 0.616 | 0.0053 | 1.43×10^-8^ | 35.07 |
| rs7684253 | C | T | -0.0392 | -0.0131 | 0.45 | 0.45 | 0.0094 | 0.162 | 0.0052 | 4.52×10^-9^ | 57.15 |
| rs78378222 | G | T | 0.1250 | -0.0736 | 0.01 | 0.02 | 0.0362 | 0.042 | 0.0229 | 1.43×10^-9^ | 29.79 |
| rs7916911 | T | G | 0.0398 | 0.0043 | 0.28 | 0.29 | 0.0105 | 0.682 | 0.0057 | 5.06×10^-9^ | 48.65 |
| rs7932866 | G | A | -0.0431 | 0.0316 | 0.16 | 0.17 | 0.0140 | 0.024 | 0.0072 | 4.16×10^-8^ | 35.69 |
| rs7996252 | C | T | -0.0289 | -0.0225 | 0.40 | 0.41 | 0.0095 | 0.018 | 0.0053 | 3.28×10^-9^ | 30.14 |
| rs8052831 | G | A | 0.0426 | 0.0161 | 0.34 | 0.35 | 0.0101 | 0.111 | 0.0055 | 4.21×10^-14^ | 60.37 |
| rs8077768 | C | T | 0.0397 | -0.0143 | 0.48 | 0.47 | 0.0110 | 0.194 | 0.0056 | 4.93×10^-8^ | 51.07 |
| rs8087942 | G | A | -0.0392 | -0.0020 | 0.33 | 0.34 | 0.0100 | 0.839 | 0.0055 | 3.18×10^-12^ | 50.99 |
| rs843215 | G | A | 0.0287 | 0.0169 | 0.47 | 0.47 | 0.0094 | 0.074 | 0.0051 | 2.38×10^-9^ | 31.02 |
| rs869432 | C | A | -0.0293 | -0.0038 | 0.42 | 0.43 | 0.0096 | 0.692 | 0.0053 | 4.11×10^-8^ | 30.43 |
| rs895219 | C | T | 0.0370 | 0.0307 | 0.30 | 0.30 | 0.0103 | 0.003 | 0.0056 | 8.25×10^-15^ | 43.82 |
| rs910187 | A | G | -0.0349 | -0.0085 | 0.37 | 0.37 | 0.0098 | 0.384 | 0.0054 | 9.32×10^-13^ | 41.64 |
| rs9295536 | A | C | -0.0355 | -0.0135 | 0.4380 | 0.4330 | 0.0091 | 0.138 | 0.0052 | 9.71×10^-13^ | 46.90 |
| rs9349379 | G | A | -0.0772 | 0.0021 | 0.4100 | 0.4040 | 0.0098 | 0.833 | 0.0053 | 2.61×10^-8^ | 210.15 |
| rs9383843 | A | C | -0.0332 | -0.0351 | 0.3530 | 0.3680 | 0.0099 | 0.000 | 0.0055 | 3.54×10^-8^ | 36.80 |
| rs9391137 | T | A | 0.0303 | 0.0081 | 0.3690 | 0.3790 | 0.0098 | 0.412 | 0.0053 | 3.74×10^-11^ | 32.21 |
| rs950570 | T | C | 0.0567 | 0.0289 | 0.0708 | 0.0630 | 0.0186 | 0.119 | 0.0100 | 1.14×10^-10^ | 32.38 |
| rs9894634 | C | T | 0.0339 | -0.0288 | 0.4030 | 0.3910 | 0.0095 | 0.002 | 0.0052 | 7.75×10^-12^ | 41.96 |

A1: effect allele; A2: reference allele; EAF: effect allele frequency; SE: standard error.

Table S7. Harmonized SNP data for two-sample MR to estimate the causal effect of genetic liability of migraine on ADHD (female-specific) .

| SNP | A1 | A2 | Beta. exposure | Beta. outcome | EAF. exposure | EAF. outcome | SE. outcome | P. outcome | P. exposure | SE. exposure |
| --- | --- | --- | --- | --- | --- | --- | --- | --- | --- | --- |
| rs10166942 | C | T | -0.091 | 0.0123 | 0.22 | 0.18 | 0.0116 | 0.291 | 1.67×10^-12^ | 0.0128 |
| rs10218452 | G | A | 0.13 | -0.0337 | 0.23 | 0.26 | 0.0132 | 0.011 | 2.17×10^-20^ | 0.0141 |
| rs1047891 | A | C | 0.064 | -0.0025 | 0.31 | 0.30 | 0.0103 | 0.812 | 2.37×10^-9^ | 0.0108 |
| rs10499112 | T | C | 0.088 | -0.0003 | 0.16 | 0.15 | 0.0125 | 0.980 | 9.16×10^-11^ | 0.0137 |
| rs10832337 | A | G | 0.067 | 0.0201 | 0.32 | 0.30 | 0.01 | 0.045 | 4.32×10^-10^ | 0.0107 |
| rs11153082 | G | A | 0.098 | -0.0060 | 0.33 | 0.34 | 0.0099 | 0.541 | 2.67×10^-20^ | 0.0106 |
| rs11172113 | C | T | -0.105 | -0.0005 | 0.41 | 0.39 | 0.0096 | 0.961 | 1.56×10^-23^ | 0.0106 |
| rs11718509 | A | G | -0.058 | -0.0072 | 0.38 | 0.42 | 0.0094 | 0.445 | 3.37×10^-8^ | 0.0104 |
| rs2078371 | C | T | 0.096 | -0.0233 | 0.12 | 0.11 | 0.0147 | 0.112 | 3.17×10^-10^ | 0.0152 |
| rs2160875 | T | C | -0.076 | 0.0092 | 0.51 | 0.54 | 0.0093 | 0.320 | 1.37×10^-13^ | 0.0103 |
| rs2274319 | C | T | -0.078 | -0.0001 | 0.65 | 0.67 | 0.0098 | 0.994 | 1.62×10^-12^ | 0.0110 |
| rs4909945 | C | T | 0.062 | -0.0030 | 0.7 | 0.69 | 0.0096 | 0.7523 | 3.89×10^-8^ | 0.0112 |
| rs7858153 | A | G | 0.107 | 0.0130 | 0.23 | 0.24 | 0.0111 | 0.2384 | 1.83×10^-19^ | 0.0118 |
| rs79545715 | C | T | -0.119 | 0.0077 | 0.08 | 0.09 | 0.018 | 0.67 | 3.63×10^-9^ | 0.0201 |
| rs8072089 | G | A | 0.074 | 0.0089 | 0.76 | 0.75 | 0.0149 | 0.5509 | 1.33×10^-9^ | 0.0121 |
| rs9349379 | G | A | -0.082 | 0.0021 | 0.4 | 0.40 | 0.0098 | 0.8326 | 6.46×10^-15^ | 0.0105 |

A1: effect allele; A2: reference allele; EAF: effect allele frequency; SE: standard error.

Table S8. Secondary sensitivity analysis -- MR estimation for causal effect of genetic liability to migraine on ADHD (IV from larger sample size GWAS (1) and female-specific GWAS (2)).

| GWAS source for exposure | exposure | outcome | method | nsnp | ci | P value | Q statistic | Q pvalue |
| --- | --- | --- | --- | --- | --- | --- | --- | --- |
| female-specific (2) | migraine | ADHD | MR Egger | 16 | 0.75 (0.57, 1) | 0.067 | 13.92 | 0.47 |
|  |  |  | Weighted median | 16 | 0.97 (0.88, 1.06) | 0.491 | 17.14 | 0.31 |
|  |  |  | Inverse variance weighted | 16 | 0.97 (0.91, 1.03) | 0.326 |  |  |
|  |  |  | Simple mode | 16 | 0.97 (0.83, 1.12) | 0.651 |  |  |
|  |  |  | Weighted mode | 16 | 0.97 (0.85, 1.12) | 0.69 |  |  |
| Primary GWAS with 23andMe (1) |  |  | Inverse variance weighted ^1^ | 109 | 1.01 (0.94, 1.09) | 0.699 |  |  |
|  |  |  | Inverse variance weighted ^2^ | 109 | 1.01 (0.97, 1.06) | 0.531 | 284.35 | 8.36×10^-18^ |
|  |  |  | MR Egger | 109 | 0.86 (0.71, 1.05) | 0.138 | 276.27 | 6.48×10^-17^ |
|  |  |  | Weighted median | 109 | 0.97 (0.9, 1.04) | 0.366 |  |  |
|  |  |  | Weighted mode | 109 | 0.95 (0.86, 1.04) | 0.253 |  |  |
|  |  |  | Simple mode | 109 | 0.96 (0.83, 1.12) | 0.594 |  |  |

^1^ random effect Inverse variance weighed model

^2^ fixed effect Inverse variance model.

Table S9. Harmonized SNP data for two-sample MR to estimate the causal effect of genetic liability to ADHD on migraine (1).

| SNP | A1 | A2 | Beta. exposure | Beta. outcome | EAF. exposure | EAF. outcome | SE. outcome | P. outcome | SE. exposure | P. exposure | F |
| --- | --- | --- | --- | --- | --- | --- | --- | --- | --- | --- | --- |
| rs10875612 | C | T | -0.0543 | 0.0035 | 0.47 | 0.50 | 0.0076 | 0.6390 | 0.0093 | 5.62×10^-9^ | 34.09 |
| rs11255890 | C | A | 0.0530 | 0.0004 | 0.40 | 0.37 | 0.0079 | 0.9640 | 0.0097 | 4.14×10^-8^ | 29.85 |
| rs114142727 | C | G | 0.2510 | -0.0166 | 0.99 | 0.98 | 0.0325 | 0.6110 | 0.0403 | 5.13×10^-10^ | 38.67 |
| rs115111850 | A | G | -0.1150 | 0.0085 | 0.95 | 0.94 | 0.0166 | 0.6100 | 0.0204 | 1.71×10^-8^ | 31.72 |
| rs11596214 | G | A | 0.0528 | 0.0010 | 0.57 | 0.60 | 0.0078 | 0.8940 | 0.0095 | 3.17×10^-8^ | 30.89 |
| rs1162202 | C | T | 0.0614 | -0.0079 | 0.61 | 0.61 | 0.0079 | 0.3210 | 0.0102 | 1.92×10^-9^ | 36.23 |
| rs1438898 | A | C | 0.0629 | 0.0128 | 0.77 | 0.75 | 0.0088 | 0.1440 | 0.0108 | 4.88×10^-9^ | 33.92 |
| rs17576773 | C | T | 0.0963 | 0.0175 | 0.88 | 0.88 | 0.0115 | 0.1290 | 0.0151 | 1.63×10^-10^ | 40.67 |
| rs17718444 | C | T | 0.0612 | 0.0131 | 0.67 | 0.69 | 0.0082 | 0.1100 | 0.0103 | 2.87×10^-9^ | 35.30 |
| rs2025286 | A | C | -0.0549 | -0.0144 | 0.55 | 0.58 | 0.0077 | 0.0611 | 0.0093 | 3.99×10^-9^ | 34.85 |
| rs2311059 | G | A | -0.0581 | -0.0131 | 0.31 | 0.32 | 0.0083 | 0.1140 | 0.0105 | 3.16×10^-8^ | 30.61 |
| rs2582895 | C | A | 0.0725 | -0.0065 | 0.62 | 0.62 | 0.0078 | 0.4050 | 0.0096 | 4.09×10^-14^ | 57.03 |
| rs2886697 | G | A | 0.0588 | -0.0045 | 0.64 | 0.63 | 0.0078 | 0.5670 | 0.0096 | 7.90×10^-10^ | 37.51 |
| rs4916723 | A | C | -0.0853 | -0.0094 | 0.57 | 0.57 | 0.0077 | 0.2220 | 0.0110 | 9.48×10^-15^ | 60.13 |
| rs4925811 | T | G | -0.0580 | -0.0178 | 0.53 | 0.53 | 0.0079 | 0.0235 | 0.0101 | 8.30×10^-9^ | 32.98 |
| rs549845 | G | A | 0.0788 | -0.0239 | 0.33 | 0.29 | 0.0083 | 0.0041 | 0.0102 | 9.03×10^-15^ | 59.69 |
| rs6082363 | T | C | 0.0703 | 0.0023 | 0.29 | 0.29 | 0.0084 | 0.7810 | 0.0101 | 4.38×10^-12^ | 48.45 |
| rs6537401 | G | A | -0.0568 | -0.0050 | 0.66 | 0.68 | 0.0081 | 0.5410 | 0.0100 | 1.40×10^-8^ | 32.27 |
| rs704061 | T | C | -0.0559 | 0.0097 | 0.56 | 0.56 | 0.0077 | 0.2050 | 0.0094 | 2.30×10^-9^ | 35.37 |
| rs73145587 | A | T | 0.1010 | -0.0204 | 0.90 | 0.89 | 0.0127 | 0.1090 | 0.0184 | 3.67×10^-8^ | 30.31 |
| rs7506904 | G | A | -0.0559 | -0.0141 | 0.37 | 0.36 | 0.0079 | 0.0765 | 0.0098 | 1.24×10^-8^ | 32.54 |
| rs7613360 | C | T | -0.0534 | -0.0161 | 0.61 | 0.61 | 0.0078 | 0.0403 | 0.0097 | 3.18×10^-8^ | 30.31 |
| rs76284431 | T | A | -0.0816 | -0.0131 | 0.84 | 0.86 | 0.0107 | 0.2230 | 0.0134 | 1.19×10^-9^ | 37.08 |
| rs76857496 | C | A | 0.0800 | 0.0179 | 0.86 | 0.86 | 0.0111 | 0.1070 | 0.0140 | 1.24×10^-8^ | 32.66 |
| rs77960 | G | A | -0.0732 | -0.0345 | 0.68 | 0.68 | 0.0082 | 0.0000 | 0.0100 | 2.46×10^-13^ | 53.59 |
| rs7844069 | T | G | 0.0552 | 0.0126 | 0.40 | 0.41 | 0.0077 | 0.1000 | 0.0095 | 6.74×10^-9^ | 33.76 |
| rs9969232 | G | A | -0.0683 | -0.0039 | 0.38 | 0.34 | 0.0080 | 0.6260 | 0.0100 | 9.98×10^-12^ | 46.65 |

A1: effect allele; A2: reference allele; EAF: effect allele frequency; SE: standard error.

Table S10. Heterogeneity and horizontal pleiotropy test for IVs (ADHD).

| exposure | outcome | method | Q statistic | Q-pvalue | beta for intercept | se for intercept | P for intercept |
| --- | --- | --- | --- | --- | --- | --- | --- |
| ADHD | migraine | Inverse variance weighted | 58.84 | 4.3×10^-4^ |  |  |  |
| ADHD | migraine | MR Egger | 55.46 | 2.4×10^-4^ | 0.01 | 0.01 | 0.229 |
